# DNMT3B splicing dysregulation mediated by SMCHD1 loss contributes to DUX4 overexpression and FSHD pathogenesis

**DOI:** 10.1126/sciadv.adn7732

**Published:** 2024-05-29

**Authors:** Eden Engal, Aveksha Sharma, Uria Aviel, Nadeen Taqatqa, Sarah Juster, Shiri Jaffe-Herman, Mercedes Bentata, Ophir Geminder, Adi Gershon, Reyut Lewis, Gillian Kay, Merav Hecht, Silvina Epsztejn-Litman, Marc Gotkine, Vincent Mouly, Rachel Eiges, Maayan Salton, Yotam Drier

**Affiliations:** ^1^ The Lautenberg Center for Immunology and Cancer Research, The Institute for Medical Research Israel-Canada, Faculty of Medicine, The Hebrew University of Jerusalem, Jerusalem 9112102, Israel.; ^2^ Department of Biochemistry and Molecular Biology, The Institute for Medical Research Israel-Canada, Faculty of Medicine, The Hebrew University of Jerusalem, Jerusalem 9112102, Israel.; ^3^ Department of Military Medicine and “Tzameret”, Faculty of Medicine, The Hebrew University of Jerusalem, Jerusalem 9112102, Israel.; ^4^ Stem Cell Research Laboratory, Medical Genetics Institute, Shaare Zedek Medical Center, Jerusalem 9103102, Israel.; ^5^ Faculty of Medicine, The Hebrew University of Jerusalem, Jerusalem 9112102, Israel.; ^6^ Department of Neurology, Hadassah Medical Organization and Faculty of Medicine, Hebrew University of Jerusalem, Jerusalem 9112002, Israel.; ^7^ UPMC University Paris 06, Inserm UMRS974, CNRS FRE3617, Center for Research in Myology, Sorbonne University,75252 Paris, France.

## Abstract

Structural maintenance of chromosomes flexible hinge domain-containing 1 (SMCHD1) is a noncanonical SMC protein and an epigenetic regulator. Mutations in SMCHD1 cause facioscapulohumeral muscular dystrophy (FSHD), by overexpressing DUX4 in muscle cells. Here, we demonstrate that SMCHD1 is a key regulator of alternative splicing in various cell types. We show how SMCHD1 loss causes splicing alterations of DNMT3B, which can lead to hypomethylation and DUX4 overexpression. Analyzing RNA sequencing data from muscle biopsies of patients with FSHD and Smchd1 knocked out cells, we found mis-splicing of hundreds of genes upon SMCHD1 loss. We conducted a high-throughput screen of splicing factors, revealing the involvement of the splicing factor RBM5 in the mis-splicing of DNMT3B. Subsequent RNA immunoprecipitation experiments confirmed that SMCHD1 is required for RBM5 recruitment. Last, we show that mis-splicing of DNMT3B leads to hypomethylation of the D4Z4 region and to DUX4 overexpression. These results suggest that DNMT3B mis-splicing due to SMCHD1 loss plays a major role in FSHD pathogenesis.

## Teaser

SMCHD1 loss drives FSHD pathogenesis by disrupting DNMT3B splicing, leading to D4Z4 hypomethylation and DUX4 overexpression.

## INTRODUCTION

Splicing of precursor mRNA (pre-mRNA) is a tightly regulated process and plays a pivotal role in shaping the transcriptome. Different exons may be spliced together in a combinatorial fashion, either including or excluding an exon from the final transcript, a phenomenon known as alternative splicing. Proper splicing is necessary for the cell’s function, and dysregulation can instigate various pathological conditions, including cancer and genetic disorders (*1*, *2*). Many factors can regulate alternative splicing, including cis-acting elements within the pre-mRNA molecule and trans-acting factors, mostly splicing factors, proteins that alter splicing by regulating splice site selection.

One major trans-acting factor regulating alternative splicing is RNA polymerase II (RNAPII). The kinetics of RNAPII elongation profoundly influences splicing outcomes; typically, slower elongation kinetics promotes exon inclusion by exposing additional splice sites (*3*). However, in some cases, slow RNAPII kinetics promotes exon exclusions (*4*), attributed to the recruitment of inhibitory splicing factors facilitated by RNAPII C-terminal domain (CTD) (*4*). Moreover, several chromatin modulators were shown to act as regulators of splicing, including the architectural regulator CCCTC-binding factor (CTCF), which can affect splicing by altering RNAPII elongation rate (*5*, *6*).

In our previous work, we conducted an unbiased high-throughput screen to identify chromatin regulators with a role in modulating alternative splicing (*7*, *8*). Our results identified 16 chromatin proteins associated with alternative splicing regulation, including the structural maintenance of chromosomes flexible hinge domain-containing 1 (SMCHD1) protein (*7*). SMCHD1 is a noncanonical SMC protein comprising an N-terminal GHKL (gyrase, Hsp90, histidine kinase, and MutL), an adenosine triphosphatase (ATPase) domain, and an SMC hinge domain that has chromatin-binding activity (*9*). Loss of SMCHD1 perturbs histone modifications, DNA methylation, CTCF occupancy, X inactivation, silencing of autosomal genes, and chromosomal interactions (*9*–*15*).

Heterozygous loss-of-function mutations in SMCHD1 cause facioscapulohumeral dystrophy type 2 (FSHD2), a late-onset progressive muscular dystrophy disease (*16*). The suggested molecular basis of FSHD2 is hypomethylation of the D4Z4 macrosatellite array, caused by SMCHD1 loss of function. This results in overexpression of the DUX4 transcription factor in muscle cells leading to inhibition of myogenesis and induction of cell death (*17*). While FSHD2 is mediated by trans factors regulating D4Z4 methylation, loss of D4Z4 methylation in FSHD1 occurs in cis by genetic contraction of the D4Z4 macrosatellite repeats (*18*). While most FSHD cases that are not attributed to D4Z4 contraction are caused by SMCHD1 mutations, FSHD has also been described in patients with mutations in the DNA methyltransferase 3 beta (DNMT3B) gene (FSHD4) and the ligand-dependent nuclear receptor interacting factor 1 (LRIF1) gene (FSHD3) (*19*, *20*). Both SMCHD1 and DNMT3B variants were identified as modifiers of disease severity in patients with FSHD1 as well (*19*, *21*, *22*). In addition, missense mutations in the ATPase domain of SMCHD1 were demonstrated to cause Bosma arhinia microphthalmia syndrome, a rare condition characterized by severe facial abnormalities, especially in the nasal area. The underlying molecular basis of the disease is not well understood but may be attributed to DUX4-mediated developmental defects (*23*–*25*).

Here, we demonstrate that SMCHD1 is a major regulator of alternative splicing. We found that SMCHD1 loss, both in mice cells and in patients with FSHD2 (but not with FSHD1), leads to global changes in splicing. Moreover, we show that aberrantly spliced genes were typically bound by SMCHD1 before SMCHD1 loss, suggesting a direct effect of SMCHD1 in regulating these genes. Last, we show that binding of SMCHD1 to abnormally spliced genes is associated with RNAPII pause. In particular, we focus on *DNMT3B* and demonstrate that SMCHD1 loss leads to preferential inclusion of exons 5, 21, and 22, and expression of the full DNMT3B isoform instead of shortened DNMT3B3ΔEx5 isoform. We show that expression of the full isoform instead of DNMT3B3ΔEx5 leads to lower methylation levels at the D4Z4 repeat array and promotes DUX4 expression, the key events causing FSHD. Thus, we propose an updated model for FSHD2 pathogenesis driven by SMCHD1-mediated alternative splicing.

## RESULTS

### Smchd1 regulates alternative splicing in mouse NSCs, embryonic fibroblasts, and embryonic stem cells

**Fig. 1.**
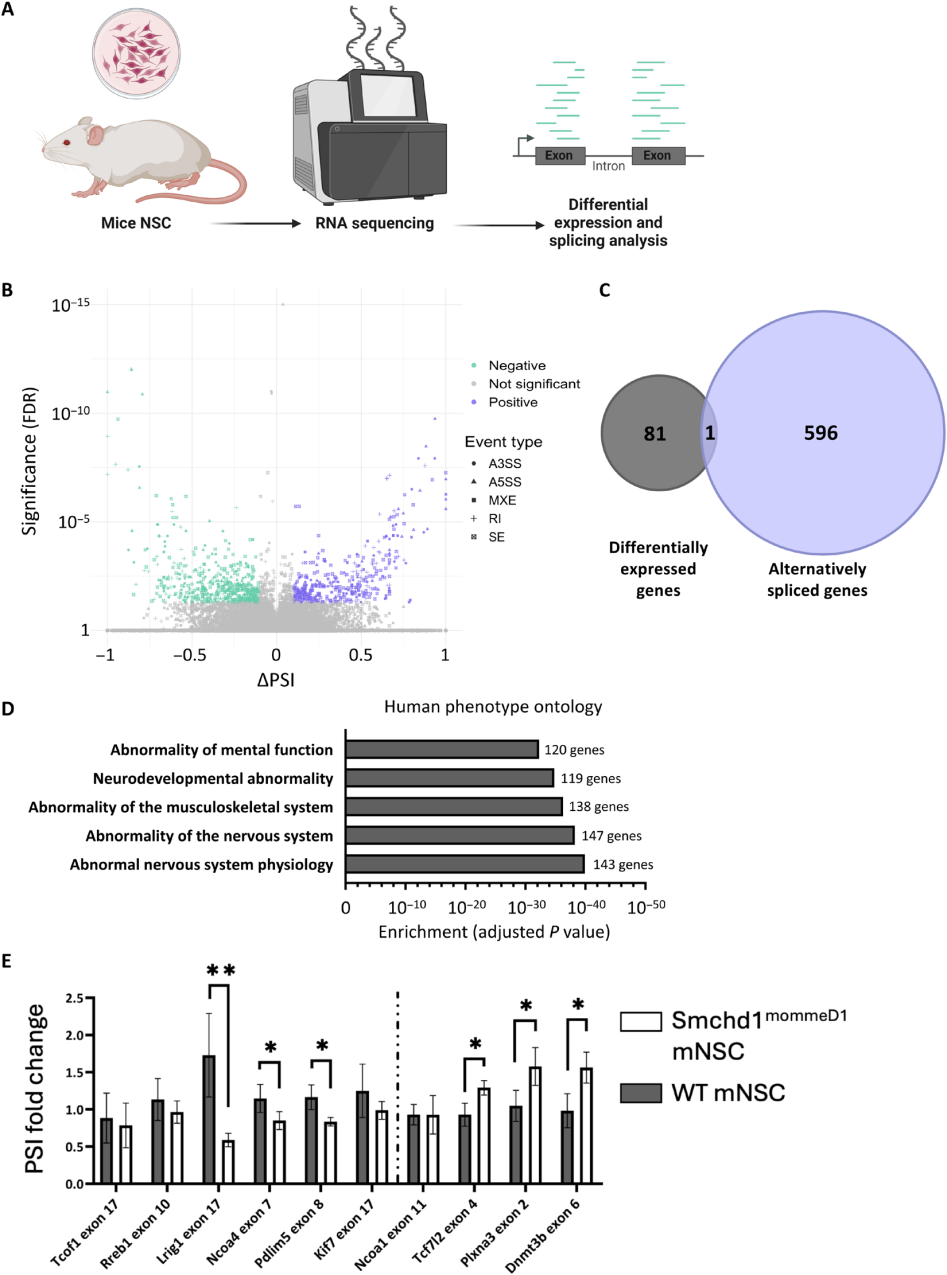
Smchd1 is a regulator of alternative splicing. (**A**) RNA was extracted from NSCs from three Smchd1 null and two WT mice and deeply sequenced. Significant alternative splicing events were detected by rMATS analysis. (**B**) Volcano plot presenting all detected alternative splicing events: *y* axis represents statistical significance (FDR), and *x* axis represents the difference of PSI. Significant events (FDR < 0.05 and |ΔPSI| > 0.1) are colored purple (ΔPSI >0.1) or green (ΔPSI < −0.1). Proportion of each alternative splicing event is presented relative to WT. (**C**) Venn diagram presenting the overlap between differentially expressed and alternatively spliced genes in Smchd1 null NSCs. (**D**) Five most significantly enriched human phenotypes (HPO) with Smchd1 null alternatively spliced genes; bars present adjusted *P* value of enrichment, and number of alternatively spliced genes are annotated next to the bar. (**E**) Real-time PCR was conducted to measure relevant splicing change and total mRNA amount. Results are shown as PSI fold change as estimated by the ratio of exon inclusion to total mRNA levels of the gene. Plots represent the mean of three Smchd1 null and four WT samples; error bars represent SD (*P < 0.05; **P < 0.01).

To explore how Smchd1 loss affects alternative splicing across different mouse cells, we started by deep sequencing RNA from neural stem cells (NSCs) sorted from Smchd1 null mice (Fig. 1A). The Smchd1^MommeD1^ mice are a previously established model with a mutation in one allele of Smchd1, resulting in Smchd1 haploinsufficiency (*9*). Here, we compared MommeD1 homozygous NSCs to wild-type (WT) controls to measure aberrant splicing in the absence of Smchd1. Differential splicing analysis identified 774 splicing events in 597 genes [Fig. 1B; false discovery rate (FDR) < 5%, |∆ percent spliced in (ΔPSI)| > 0.1, rMATS], 49% of the splicing events due to Smchd1 loss are exon-skipping events (378 events) (fig. S1A and table S1). The other 51% is composed of different splicing events including mutually exclusive exons (38 events), intron retention (133 events), alternative 3′ splice site (130 events), and alternative 5′ splice site (95 events). Fifty-nine % of these events presented higher inclusion in Smchd1^MommeD1^ samples, and 41% presented higher exclusion, suggesting that Smchd1 is regulating splicing in both directions. We identified 82 differentially expressed genes (DESeq2, FDR < 5%), and only one gene was both differentially expressed and alternatively spliced (Fig. 1C). Gene set enrichment analysis for differentially spliced genes in the NSCs revealed significant enrichment for phenotypes related to muscular dystrophy. Twenty-three % of mis-spliced genes (138 of 597 genes, FDR < 5.3 * 10^−37^) are related to the “Abnormality of the musculoskeletal system” Human Phenotype Ontology (HPO) (Fig. 1D and table S2).

To validate the splicing analysis, we chose 10 leading candidate genes that were significantly mis-spliced and matched either an HPO annotation of “abnormal muscle physiology” or a Gene Ontology annotation of “anatomical structure development”. We performed quantitative polymerase chain reaction (qPCR) on RNA from the same cells and found 6 of the 10 genes to be significantly mis-spliced (*P* < 0.05, Student’s *t* test) (Fig. 1E). Of those, only Kif7 and Dnmt3b demonstrated a change in total expression levels (fig. S1B).

To explore the splicing deregulation by Smchd1 loss in additional mouse cells, we reanalyzed available RNA sequencing (RNA-seq) data in mouse embryonic fibroblasts (MEFs) and mouse embryonic stem cells (mESCs). Our analysis found 3398 alternative splicing events in 2114 genes in Smchd1^MommeD1^ homozygous mutant female MEFs (*12*) (rMATS, FDR < 5%, |ΔPSI| > 0.1; fig. S1C and table S1) and 924 events in 618 genes in Smchd1-KO mESCs (fig. S1D and table S1) (*26*). Gene set enrichment analysis for differentially spliced genes in MEFs and mESCs revealed significant enrichment for phenotypes related to muscular dystrophy (fig. S1, E and F, and table S2). The identity of the alternatively spliced genes may change between cell types, but many of them are shared between two or more cell types (*P* < 0.00001, Fisher’s exact test; fig. S1G). Genes whose splicing is regulated by Smchd1 tend to be related to muscle dystrophy, even in unrelated cell types, demonstrating the potential of Smchd1-mediated splicing to affect muscle dystrophy, which can be unleashed in muscle cells. Together, these findings indicate an important role for Smchd1 as a regulator of alternative splicing across several different cell types independently of its role in gene expression regulation.

### Smchd1 binding is enriched at mis-spliced exons

**Fig. 2.**
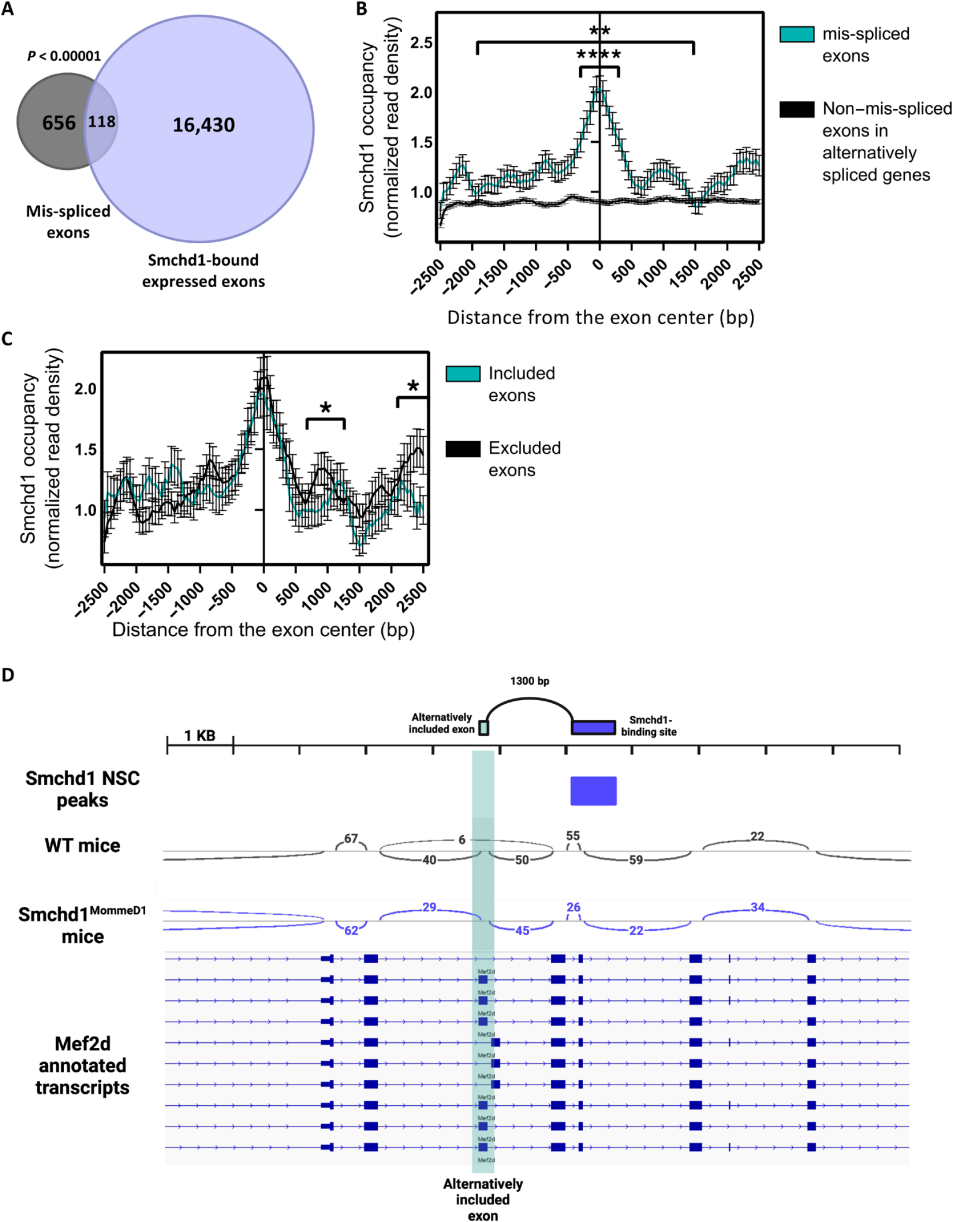
Smchd1 binding is enriched downstream of its regulated excluded exons. (**A** to **D**) Reanalysis of GFP ChIP-seq in primary mouse NSCs with endogenous Smchd1-GFP fusion protein; analysis was limited to expressed genes only [transcripts per million (TPM) > 1]. (A) Venn diagram presenting the overlap of mis-spliced exons and exons with nearby (<5 kbp) Smchd1-binding site. (B) Aggregation plot depicting the average normalized Smchd1 occupancy at and near exons mis-spliced (turquoise) or non–mis-spliced exons (black) in alternatively spliced genes, showing stronger binding of Smchd1 near mis-spliced exons (**P < 0.01, ****P < 0.0001). *X* axis represents bins of size 50 bp around the center of the exon. (C) Aggregation plot depicting the average normalized Smchd1 occupancy at exons differentially included or excluded in Smchd1 WT NSCs compared to Smchd1 null NSCs (*P < 0.05). *X* axis represents bins of size 50 bp around the center of the exon. (D) Genome browser view of the Mef2d alternatively spliced junctions presented by sashimi plots; arcs denote splice junctions quantified in spanning reads. Mutually exclusive alternatively included exon is highlighted in turquoise. Refseq transcripts are presented as a reference.

Next, we wanted to explore how Smchd1 regulates splicing. Since Smchd1 is a chromatin factor, we started by exploring the chromatin landscape at its splicing targets. We began by investigating whether Smchd1 directly binds mis-spliced exons. To this end, we analyzed available Smchd1–green fluorescent protein (GFP) chromatin immunoprecipitation sequencing (ChIP-seq) data from NSCs of WT mice (*10*). First, we assessed Smchd1 binding in the vicinity (5 kb) of mis-spliced exons and compared them to all exons expressed in either WT or Smchd1^MommeD1^ NSCs, regardless of whether they are mis-spliced or not. We found that Smchd1 binds 118 (15%) of mis-spliced exons, compared to 7% of all expressed exons (Fig. 2A; *P* < 0.00001, Fisher’s exact test). Overall, Smchd1 binds mis-spliced exons 2.25-times more often than all expressed exons. Moreover, we compared Smchd1 binding in mis-spliced exons to other exons in the same genes that are not alternatively spliced and found that Smchd1 binds the mis-spliced exons 1.41-times more often (Fig. 2B; *P* < 0.0003, Fisher’s exact test).

We found that Smchd1 preferentially binds downstream to exons that are included in Smchd1 null cells but excluded when bound by Smchd1 in WT cells (“excluded exons”) (*P* < 0.05, Wilcoxon test) (Fig. 2C), for example, exon 5 of *Mef2d*, a member of the myocyte-specific enhancer factor 2 (Mef2) family involved in muscle cell differentiation and development (*27*). Exon 5 is adjacent to an Smchd1-binding site and skipped in WT mice but included in Smchd1^MommeD1^ mice (Fig. 2D). We repeated the analysis for another available NSC Smchd1-GFP ChIP-seq dataset (*28*) and found that Smchd1 binds 26% of mis-spliced exons, 3.57-times more compared to all expressed exons (*P* < 0.00001, Fisher’s exact test) and specifically binds 1.36-times more to excluded exons (*P* < 0.028, Student’s *t* test; fig. S2, A to C). Moreover, we repeated this analysis for MEFs, using published Smchd1-binding data in MEFs (*12*). We observed a similar association with splicing: Mis-spliced exons had 1.97-times more Smchd1-binding sites than all expressed exons (*P* < 0.00001, Fisher’s exact test), and Smchd1 preferentially binds downstream of excluded exons (fig. S2, D and E). In addition, we repeated the analysis for a 1-kb window around the exon and found similar enrichment of Smchd1 in all datasets. Smchd1 binds 1.5-times and 2.4-times more to alternatively spliced exons compared to all expressed exons in the NSC datasets (*P* < 0.016 and *P* < 0.00001, Fisher’s exact test) and 1.3-times more (*P* < 0.004, Fisher’s exact test) in MEFs. This significant overlap between Smchd1 binding and alternatively spliced exons suggests a direct effect of Smchd1 binding on alternative splicing. The relatively modest effect sizes suggest that some genes may be also indirectly affected, and some Smchd1-binding sites may not yield a detectible difference in splicing. Overall, our analysis demonstrates that the binding of Smchd1 is enriched downstream of excluded exons, suggesting that Smchd1 loss leads to aberrant inclusion of the exons.

### Smchd1 binding correlates with RNAPII stalling at mis-spliced exons

**Fig. 3.**
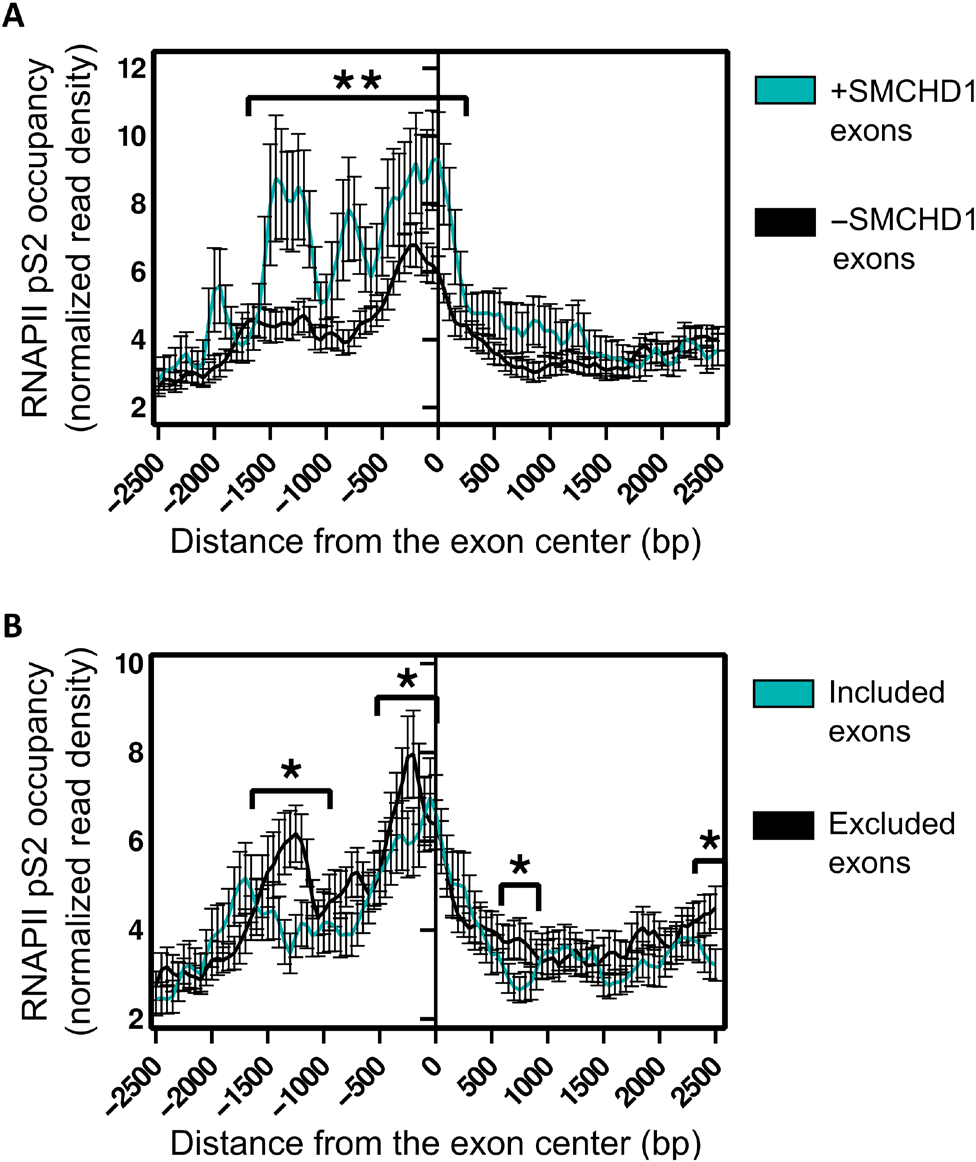
Smchd1 binding is associated with slow elongation rate of RNAPII. (**A** and **B**) Aggregation plots depicting the average normalized phospho-Ser2 levels of RNAPII in C2C12 cells at and near mis-spliced exons differentially bound by Smchd1 (A) and at and near exons differentially included or excluded in Smchd1 WT NSCs (B). *X* axis represents bins of size 50 bp around the center of the exon (*FDR < 0.05; **FDR < 0.01).

Since RNAPII elongation rate can regulate alternative splicing, we next asked whether Smchd1 binding is associated with RNAPII elongation. To address this, we reanalyzed ChIP-seq data of phosphoserine 2 of RNAPII CTD (pSer2), a marker of elongating RNAPII, from C2C12 mouse myoblasts (*29*). Enrichment of pSer2 marks a slow elongation rate or stalling of RNAPII (*30*). We compared the RNAPII pSer2 signal between mis-spliced exons with and without Smchd1 binding. Our analysis revealed a significant enrichment of RNAPII pSer2 in the exons bound by Smchd1 (Fig. 3A). We repeated the analysis for Smchd1-binding sites identified in a different NSC dataset (*31*) or comparing Smchd1-binding sites in MEFs (*12*) to RNAPII ChIP-seq data from MEFs (*29*) and also found a significant enrichment of RNAPII pSer2 or RNAPII at Smchd1-bound mis-spliced exons (fig. S3, A and B). Moreover, we compared RNAPII pSer2 signal between included or excluded exons and found a specific enrichment at excluded exons (*P* < 0.05, Wilcoxon test; Fig. 3B). Together, these results suggest that Smchd1 binding in mis-spliced exons is correlated with RNAPII stalling and exon exclusion.

### Differential splicing in FSHD2 patients with SMCHD1 mutation

**Fig. 4.**
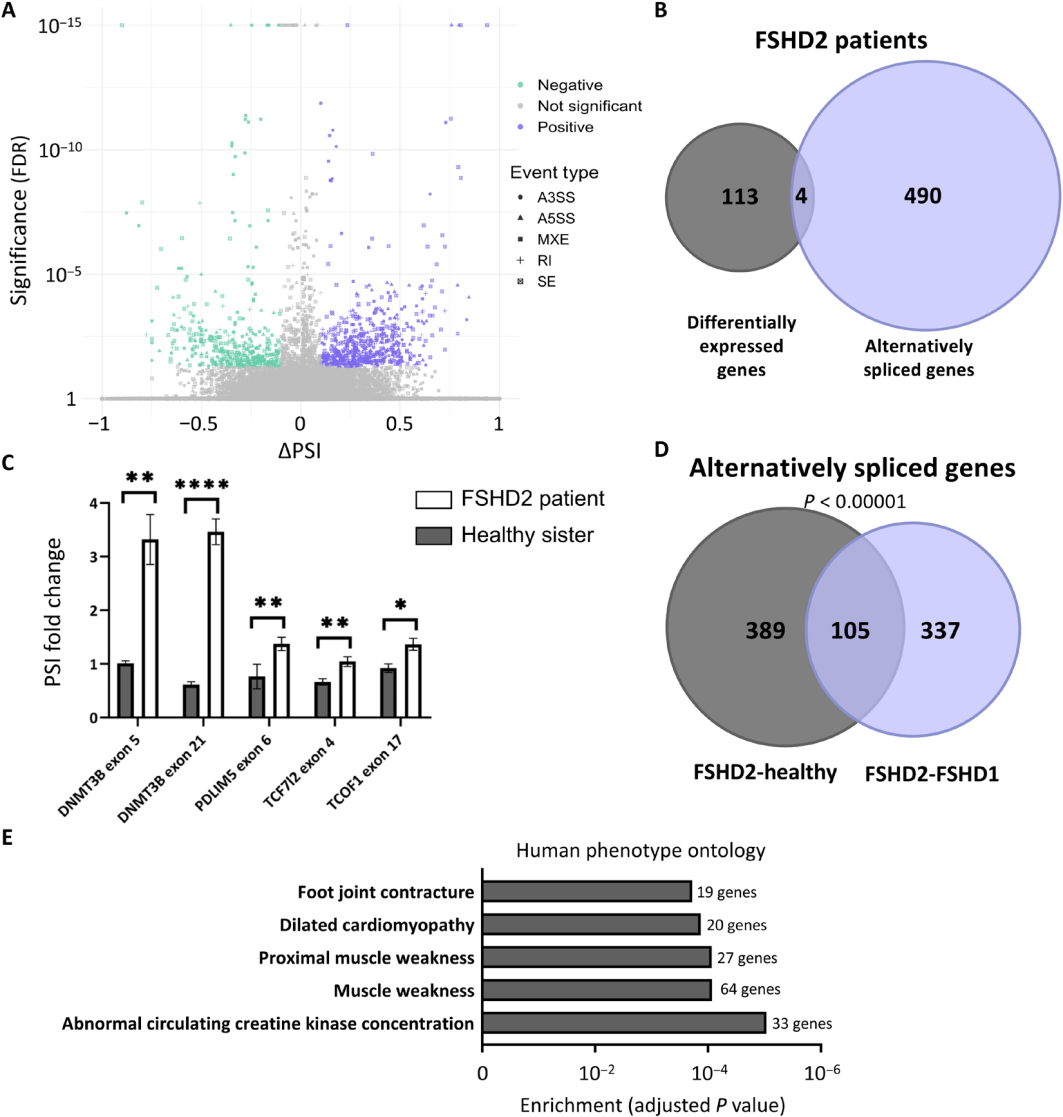
SMCHD1 is a regulator of alternative splicing in patients with FSHD. (**A** and **B**) Significant alternative splicing in muscles of patients with FSHD2 revealed by rMATS analysis of RNA-seq data from healthy individuals and patients with FSHD1 and with FSHD2 (*32*). (A) Volcano plot presenting all detected alternative splicing events between muscles of patients with FSHD2 and healthy individuals. *Y* axis represents statistical significance (FDR), and *x* axis represents PSI difference. Significant events (FDR < 0.05 and |ΔPSI| > 0.1) are colored purple (ΔPSI >0.1) or green (ΔPSI < −0.1). (B) Venn diagram presenting the overlap between differentially expressed and alternatively spliced genes in patients with FSHD2. (**C**) RNA was extracted from lymphoblasts of a female patient with FSHD2 and her healthy sister. Real-time PCR was conducted to measure the relevant splicing event and total mRNA amount. Results are shown as PSI fold change as estimated by the ratio of exon inclusion to total mRNA levels of the gene. Values represent averages of three biological replicates performed in three technical replicates (*P < 0.05; **P < 0.01; ****P < 0.0001). (**D** and **E**) Significant alternative splicing in muscles of patients with FSHD2 revealed by rMATS analysis of RNA-seq data from healthy individuals and patients with FSHD1 and FSHD2. (D) Venn diagram presenting the overlap of alternative splicing events when patients with FSHD2 are compared to those with FSHD1 or to healthy individuals. (E) Five most significantly enriched human phenotypes (HPO) with FSHD2 alternatively spliced genes; bars present adjusted *P* value of enrichment; number of alternatively spliced genes are annotated next to bar.

To test whether splicing regulation by SMCHD1 is relevant to FSHD, we compared alternative splicing between patients with SMCHD1-mutated FSHD2 and healthy controls by available RNA-seq data (*32*). We found differential expression of 117 genes (FDR < 5%, DESeq2) and mis-splicing of 494 genes (FDR < 5%, |ΔPSI| > 0.1, rMATS). A total of 776 mis-splicing events were identified, 46% of them are exon-skipping events (355 events) (Fig. 4A, fig. S4A, and table S1). The other 54% is composed of different alternative splicing events including mutually exclusive exons (197 events), intron retention (72 events), alternative 3′ splice site (66 events), and alternative 5′ splice site (86 events). Only four genes were identified as both differentially expressed and alternatively spliced, again supporting the hypothesis that SMCHD1 independently regulates gene expression and splicing (Fig. 4B). To validate the mis-splicing in FSHD2 patient cells, we generated lymphoblasts from a patient with a pathogenic missense mutation in SMCHD1 ATPase domain (p.Leu194Phe) and her healthy sister (fig. S4, B to D), and this mutation was previously recognized to cause FSHD2 (*33*). SMCHD1 expression on both mRNA and protein levels of the patient was lower, consistent with observations reported in the literature for this mutation (*34*) (fig. S4, B to D). Our qPCR results found a change in splicing in all five splicing events, and no significant change was detected in total mRNA levels (Fig. 4C and fig. S4E).

In principle, these splicing changes may reflect changes between dystrophic muscle and healthy muscle and not changes due to the SMCHD1 mutations. To rule out this option, we repeated the analysis but compared patients with FSHD2 to those with FSHD1. This analysis found 679 splicing events in 442 genes (FDR < 5%, |ΔPSI| > 0.1, rMATS), with 24% of the genes (105) also detected in the previous analysis (*P* < 0.00001, Fisher exact test; Fig. 4D), suggesting that, indeed, the cause of the splicing differences is the SMCHD1 mutation. While patients with FSHD1 and with FSHD2 have similar clinical presentation, the observed splicing changes may reflect the difference in the molecular pathogenesis of the diseases or could reflect side effects of the global disruption of splicing regulation by SMCHD1. Overall, these results reveal significant mis-splicing in patients with SMCHD1-mutated FSHD2, suggesting global splicing disruption in patients’ muscle cells due to loss of SMCHD1.

We next asked how splicing alteration by SMCHD1 can contribute to the phenotype of FSHD2. To this end, we explored the function of genes regulated in splicing by gene set enrichment analysis, compared to all expressed [transcripts per million (TPM) >1] genes in muscle tissue. Twenty-six % [130 genes of 494, FDR < 0.0003) of the genes mis-spliced in patients with FSHD2 are associated with “abnormality of the musculature” annotation by the HPO (*35*)], suggesting that many genes mis-spliced because of SMCHD1 mutations in FSHD2 are associated with abnormal muscle function. The top significant enriched human phenotype terms included many annotations associated with muscular dystrophy that are related to FSHD2 pathology (Fig. 4E and table S2). Analysis of genes differentially spliced between FSHD2 and FSHD1 yielded similar results—113 genes of 442 alternatively spliced (25%) were associated with abnormality of the musculature (FDR < 0.022; table S2), demonstrating that this is indeed due to SMCHD1 mutations and not due to other FSHD-related mis-splicing. To further support this claim, we repeated the analysis to compare patients with FSHD1 to healthy controls, and indeed, in this case, no significant enrichment of any muscle-related phenotypes was detected, showing that the excessive mis-splicing of genes related to musculature abnormalities is not simply due to muscular dystrophy. Gene set enrichment analysis of differentially expressed genes between either FSHD2 and healthy controls or between FSHD2 and FSHD1 revealed no enrichment for FSHD-related phenotypes.

### DNMT3B splicing is disrupted by SMCHD1 loss in mouse and human cells

Our analysis revealed that *DNMT3B* splicing is regulated by SMCHD1 in human patients with FSHD2 (Fig. 4C), mouse NSCs (Fig. 1E and fig. S5A), MEFs (rMATS FDR < 0.0004), and mouse ESCs (rMATS FDR < 0.003). In the human patient lymphoblasts, *DNMT3B* presented significant splicing changes in exons 5 and 21-22 and in mouse NSCs in the corresponding mouse exons 6 and 20-21. However, in MEFs, exon 16 is alternatively spliced (FDR < 0.0004, ΔPSI = 0.89), and in mESCs, exon 2 is alternatively spliced (FDR < 0.0003, ΔPSI = 0.06) as well as exon 3 (FDR < 0.015, ΔPSI = 0.09). As DNMT3B levels are very low in adult muscle tissue (0.4 to 0.5 TPM), we were unable to confidently call differential splicing of DNMT3B from the FSHD patients’ single-end RNA-seq data.

**Fig. 5.**
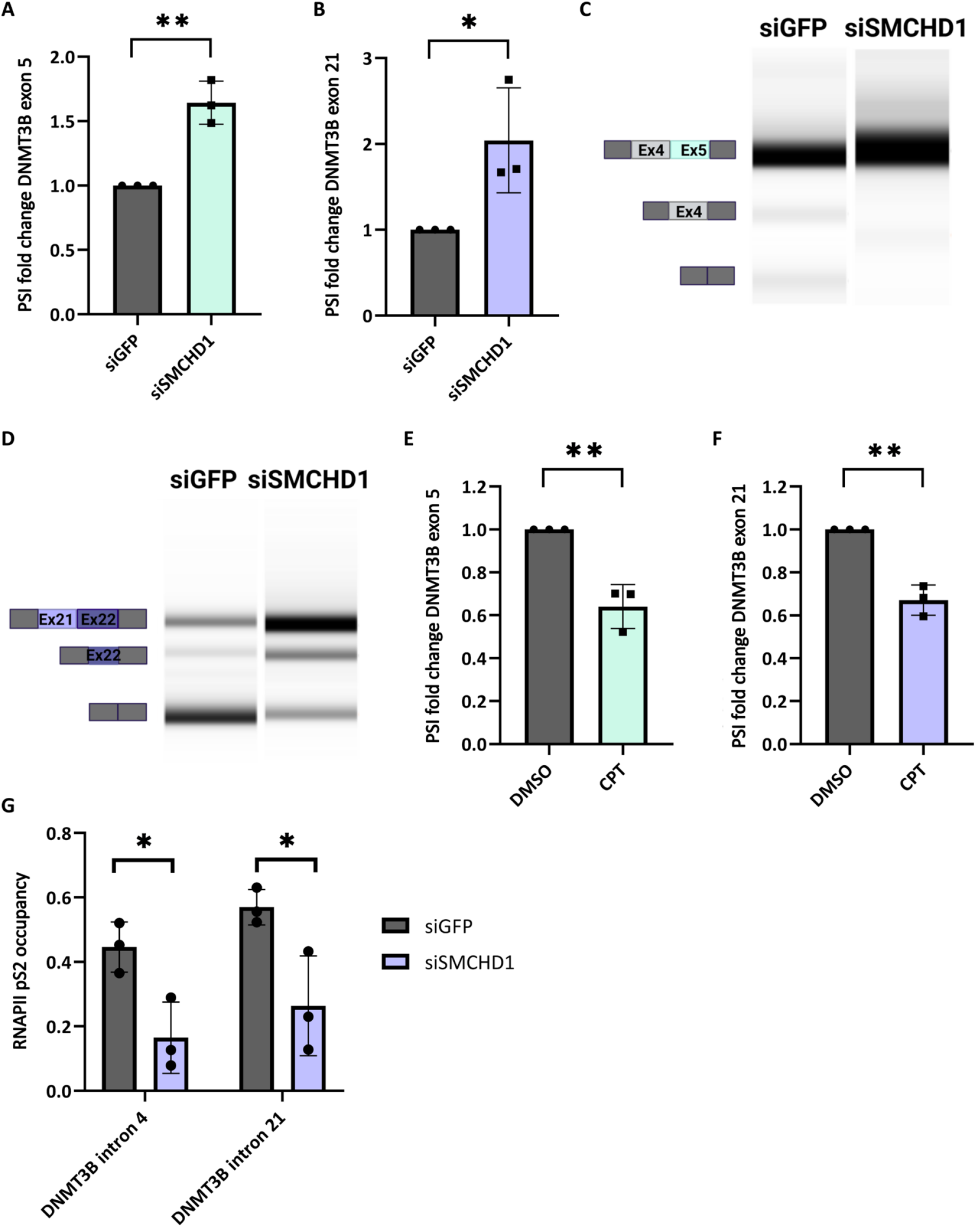
DNMT3B exons 5 and 21 are regulated by SMCHD1 and RNAPII stalling. (**A** to **D**) HCT116 cells were transfected with siRNA targeting SMCHD1 and GFP as negative control for 72 hours. Total RNA was extracted and analyzed by real-time PCR for DNMT3B exon 5 (A) and exon 21 (B) inclusion relative to DNMT3B total mRNA amount. PSI fold change was estimated by the ratio of exon inclusion to total DNMT3B mRNA levels. Data represent mean ± SD of three independent experiments performed in triplicates. (C) Semiquantitative PCR was conducted for exons 4 and 5. (D) Semiquantitative PCR for exons 21 and 22 was followed by sequencing of the indicated bands. Sequencing results informed the schematic representation of the exons. (**E** and **F**) HCT116 cells were treated with 6 μM CPT or dimethyl sulfoxide (DMSO) as negative control, for 6 hours. Total RNA was extracted and analyzed by real-time PCR for DNMT3B exon 5 (E) and exon 21 (F) inclusion relative to DNMT3B total mRNA amount. PSI fold change was calculated by dividing exon inclusion in DNMT3B total mRNA amount. Values represent mean ± SD of three independent experiments performed in triplicates (*P < 0.05; **P < 0.01) (paired Student’s *t* test). (**G**) HCT116 cells were transfected with siRNA targeting SMCHD1 and GFP as negative control, followed by immunoprecipitation for RNAPII pSer2. Real-time PCR was conducted for DNMT3B introns 4 and 21 relative to input. Values represent averages of three technical replicates; error bars represent SD. (*P < 0.05) (Student’s *t* test).

* DNMT3B* is known to have dozens of alternatively spliced isoforms with distinct functions (*36*). *DNMT3B* exons 21 and 22 encode part of the C-terminal catalytic domain of the protein, while the exon 5 region does not contain any known functional domains. DNMT3B3ΔEx5 isoform is known to be associated with increased DNA binding affinity and enhanced cell growth (*36*). Mutations of *DNMT3B* in FSHD are associated with D4Z4 hypomethylation and with high levels of *DUX4* expression (*19*). Specifically, a mutation in *DNMT3B* C-terminal catalytic domain was shown to cause FSHD (*19*). Alternative splicing of *DNMT3B* may affect D4Z4 methylation and thus contribute to disease development. DNMT3B-mediated regulation of DNA methylation occurs mostly during differentiation, and, therefore, loss of SMCHD1 is likely detrimental at these stages. To study *DNMT3B* splicing in partially differentiated cells, we chose HCT116 cells, a colon cancer stem–like cell line, where DNMT3B was shown to be expressed, active, and to play a role in DNA de novo methylation and maintenance (*37*). To validate SMCHD1-mediated alternative splicing of *DNMT3B* in human cells, we knocked down SMCHD1 by small interfering RNA (siRNA) in HCT116 cells (fig. S5B), a cancer cell line with expressed and active DNMT3B. qPCR analysis showed a 1.6-fold increase in the inclusion of exon 5 (*P* < 0.05, Student’s *t* test) and a twofold increase in the inclusion of exon 21 (*P* < 0.01, Student’s *t* test) (Fig. 5, A and B). Semiquantitative PCR analysis confirmed these results (Fig. 5, C and D, and fig. S5, C and D). Overall, this demonstrates that *DNMT3B* alternative splicing is indeed regulated by SMCHD1.

Our previous results suggested that SMCHD1 regulates alternative splicing by binding to the vicinity of the alternative exon and slowing RNAPII elongation rate. To investigate whether *DNMT3B* alternative splicing is mediated by slower RNAPII, we treated HCT116 cells with camptothecin (CPT), a topoisomerase inhibitor that slows RNAPII elongation. We used a low dose of 6 μM CPT to slow down RNAPII without stopping it while also avoiding any potential DNA damage. Our results show a 70% reduction in the total mRNA amount of DNMT3B and a 35% reduction in exon 5 and 21 inclusion following treatment with CPT (*P* < 0.01, Student’s *t* test) (Fig. 5, E and F, and fig. S5E), suggesting that RNAPII stalling indeed promotes exclusion of SMCHD1-regulated exons at *DNMT3B* gene. To investigate the potential regulation of RNAPII stalling at *DNMT3B* by SMCHD1, we conducted a ChIP assay for RNAPII pSer2 in HCT116 cells after SMCHD1 knockdown using siRNA. Subsequently, we performed qPCR analysis with primers targeting regions adjacent to RNAPII-stalling sites within the *DNMT3B* gene, specifically intron 4 and intron 21. The qPCR results revealed a significant reduction in RNAPII pSer2 enrichment following SMCHD1 knockdown, with a 63% decrease at intron 4 (*P* < 0.02, Student’s *t* test) and a 54% decrease at intron 21 (*P* < 0.02, Student’s *t* test) (Fig. 5G). These findings collectively indicate a dependence of RNAPII stalling at the *DNMT3B* gene on SMCHD1.

### Identification of splicing factors regulating the alternative splicing of DNMT3B

Our previous findings suggest that SMCHD1 is promoting exon exclusion by RNAPII stalling. We hypothesize that RNAPII stalling by SMCHD1 promotes exclusion by recruiting splicing factors. To predict which splicing factors may be involved, we performed RNA binding proteins motif analysis on exons excluded by SMCHD1. To this end, we compared excluded exons and their flanking 10-kb sequences to those of included exons in mouse NSCs and human patients with FSHD2. Our analysis found 35 significantly enriched motifs in human and 17 in mice. Five motifs were found enriched in both datasets: RBFOX1, CPO, MSI, RBM5, and SRSF10 (fig. S6A). Next, we tested whether these motifs are also specifically enriched in paused RNAPII sites proximal to alternatively spliced exons with Smchd1 binding compared to those that lack Smchd1 binding and found significant enrichment only for the RBM5 motif (enrichment ratio 1.77, *P* < 0.015).

**Fig. 6.**
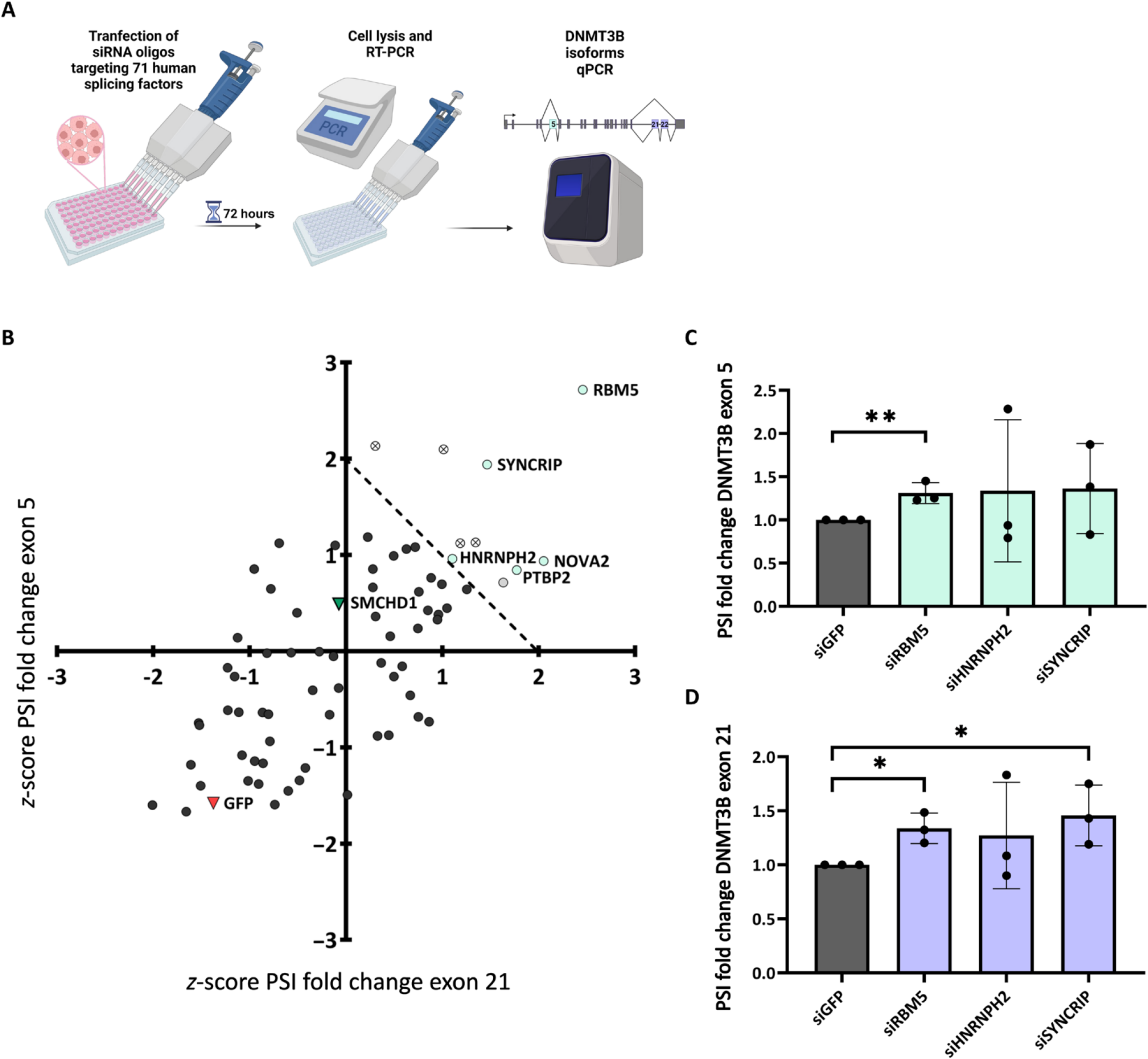
* DNMT3B* exon 5 and 21 are regulated by RBM5. (**A**) HCT116 cells were transfected with siRNA targeting 71 human splicing factors, SMCHD1 as a positive control and GFP as negative control for 72 hours. Total RNA was extracted and analyzed by real-time PCR for *DNMT3B* exon 5 and exon 21. *Z* scores were calculated for *DNMT3B* exon 5 and exon 21 PSI fold change, as *DNMT3B* exon inclusion/DNMT3B total mRNA. PSI fold change values were normalized to siGFP before *z*-score calculation to normalize variance between each experiment. (**B**) Scatter plot shows *z* scores of PSI fold change of exon 5 versus exon 21; the red triangle represents GFP (negative control); the green triangle represents SMCHD1 (positive control); gray circles represent a lowly expressed factor; crossed circles represent hits that are inconsistent between repeats; and blue circles represent splicing factor hits. Data represent two independent experiments performed in three technical replicates. (**C** and **D**) HCT116 cells were transfected with siRNA targeting each of the splicing factor hits and GFP as a negative control. Total RNA was extracted and analyzed by real-time PCR for *DNMT3B* exon 5 (C) and exon 21 (D) inclusion relative to DNMT3B total mRNA amount, normalized to negative control. Values represent averages of three independent experiments performed in triplicates; error bars represent SD. (*P < 0.05, **P < 0.01) (Student’s *t* test).

To experimentally identify potential splicing factors cooperating with SMCHD1, we performed an unbiased siRNA screen with the use of *DNMT3B* alternative splicing as readout. Specifically, we used a library of siRNA oligos directed to the 71 human splicing factors [as described in SpliceAid-F (*38*)] in HCT116 cells (Fig. 6A). We monitored alternative splicing of *DNMT3B* exons 5 and 21 using qPCR. As SMCHD1 is regulating the alternative splicing of both exons 5 and 21 of *DNMT3B*, we expected that a splicing factor working with SMCHD1 will regulate both events. *Z* scores were calculated for the average PSI of exons 5 or 21 separately. Splicing factors for which both *z* scores are higher than the positive control (siSMCHD1) and for which the sum of both *z* scores was higher than 2 were considered for downstream analysis. The screen identified six factors: RBM5, SYNCRIP, HNRNPH2, NOVA2, PTBP2, and ELAVL3 (Fig. 6B). Of those six factors, we filtered out factors with expression level below detection rate and eventually found five hits: RBM5, SYNCRIP, HNRNPH2, NOVA2 and PTBP2 (fig. S6B). To validate these hits, we conducted a secondary screen knocking down each splicing factor by siRNA in HCT116 cells and tested the inclusion of DNMT3B exons while monitoring the knock-down level of each splicing factor (fig. S6C). The silencing of PTBP2 and NOVA2 was unsuccessful in the secondary screen, and we can assume that it was similarly unsuccessful in the original screen. Therefore, we excluded them from further analysis. qPCR analysis showed a significant increase in the inclusion of DNMT3B exons 5 and 21 only for RBM5 (31 and 34% increase respectively, *P* < 0.005 and *P* < 0.017, Student’s *t* test) (Fig. 6, C and D). Overall, motif enrichment and splicing factor screen suggest RBM5 as a potential ally of SMCHD1 in its alternative splicing regulation.

### DNMT3B alternative splicing is regulated by SMCHD1 via RBM5

**Fig. 7.**
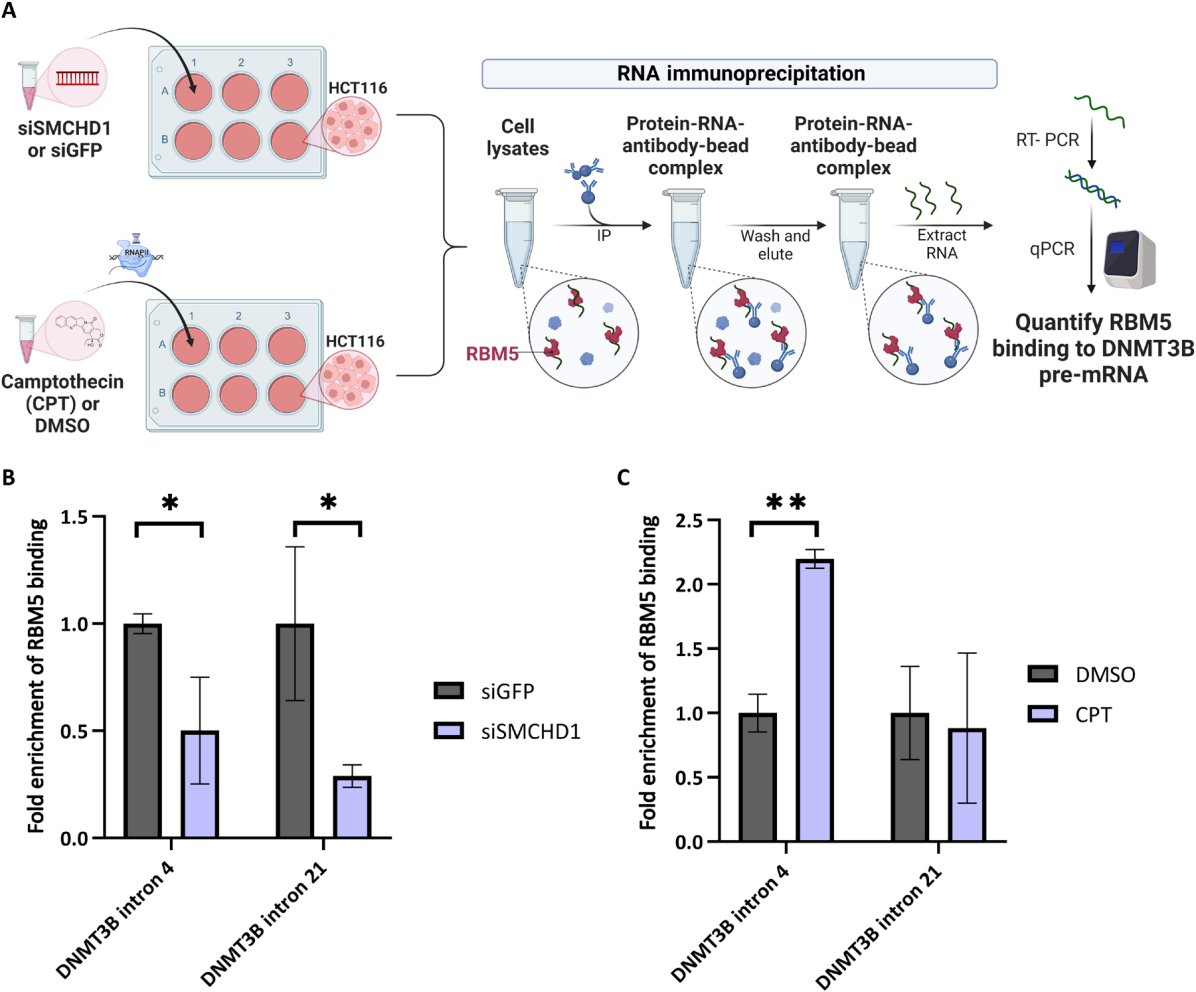
RBM5 binding to DNMT3B exon 5 and 21 is SMCHD1 dependent. (**A**) RNA immunoprecipitation of RBM5 in HCT116 cells following silencing of SMCHD1 or CPT treatment. (**B**) HCT116 cells were transfected with siRNA targeting SMCHD1 and GFP as negative control for 72 hours. Bars show real-time PCR results for DNMT3B introns 4 and 21 relative to input. (**C**) HCT116 cells were treated with either vehicle (DMSO) or 6 μM CPT for 6 hours. Bars show real-time PCR results for DNMT3B introns 4 and 21 relative to input. Values represent averages of three experiments performed in triplicates; error bars represent SD. (*P < 0.05, **P < 0.01) (paired Student’s *t* test).

We hypothesize that SMCHD1 binding to the DNA stalls RNAPII to allow RBM5 binding to pre-mRNA. To check our hypothesis, we silenced SMCHD1 in HCT116 and measured the binding of RBM5 using RNA-IP to DNMT3B pre-mRNA while monitoring the expression of RBM5 (Fig. 7A and fig. S6D). Monitoring DNMT3B introns 4 and 21, we found that silencing of SMCHD1 led to a reduction of 50 and 70% in RBM5 binding, respectively (*P* < 0.013 and *P* < 0.017, Student’s *t* test) (Fig. 7B). This result suggests that SMCHD1 is a regulator of RBM5 binding to DNMT3B’s pre-mRNA.

To test whether RNAPII stalling mediates RBM5 binding, we performed RNA-IP following CPT treatment and found a twofold increase in RBM5 binding to DNMT3B intron 4 (*P* < 0.004, Student’s *t* test), while there was no change in RBM5 binding to DNMT3B intron 21 (Fig. 7C). RBM5 total expression level remained unchanged in CPT-treated cells (fig. S6D). Overall, these results support the hypothesis that the RBM5 and SMCHD1 proteins functionally interact in the regulation of DNMT3B alternative splicing, and that RBM5 recruitment to DNMT3B exon 5 is affected by RNAPII stalling.

### DNMT3B alternative splicing promotes D4Z4 hypomethylation and DUX4 expression

**Fig. 8.**
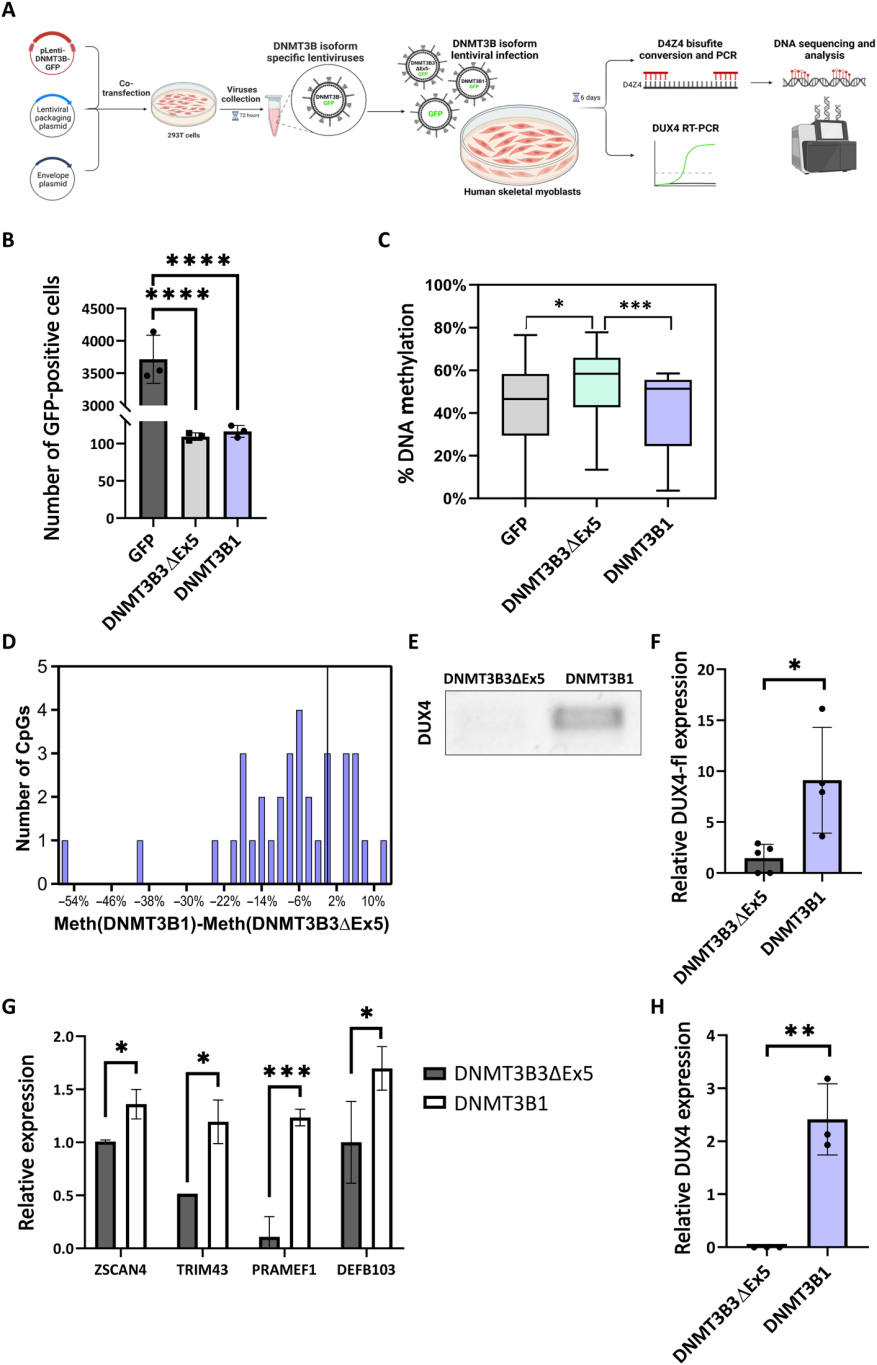
DNMT3B1 isoform reduces methylation at the D4Z4 region leading to increase of DUX4 expression. (**A**) Human skeletal myoblasts were infected with lentiviruses containing empty GFP, GFP-DNMT3B3ΔEx5, or GFP-DNMT3B1. D4Z4 methylation was quantified by bisulfite PCR sequencing and *DUX4* expression by semiquantitative PCR and real-time PCR. (**B**) Quantification of GFP expressing cells in infected myoblasts (****P < 0.0001). (**C** and **D**) DNA was isolated from human skeletal myoblasts infected with each of the isoforms and converted with bisulfite. PCR products of the D4Z4 region were sequenced, and methylation levels in each CpG were assessed using Biscuit. Boxplot (C) presents the methylation level in each isoform-specific cell line, and histogram (D) presents the difference between methylation levels measured at the D4Z4 region in DNMT3B1 and DNMT3B3ΔEx5 expressing cells. (**E** to **G**) RNA was extracted from human skeletal myoblast cells with the DNMT3B3ΔEx5 and DNMT3B1 isoforms. (E) Semiquantitative PCR was conducted for *DUX4* mRNA level. (F) Real-time PCR was conducted for DUX4-fl mRNA level. DUX4-fl mRNA level was quantified relative to *CycloA* reference gene. Values represent averages of four technical replicates; error bars represent SD. (*P < 0.05) (Student’s *t* test). (G) Real-time PCR was conducted for DUX4 targets *ZSCAN4*, *TRIM43*, *PRAMEF1*, and *DEFB103*. Values represent averages of three technical replicates; error bars represent SD. (*P < 0.05, **P < 0.01, ***P < 0.001) (Student’s *t* test). (**H**) hESCs were infected with lentiviruses containing empty GFP, GFP-DNMT3B3ΔEx5, or GFP-DNMT3B1. RNA was extracted, and real-time PCR was conducted for DUX4 mRNA level. DUX4 mRNA level was quantified relative to *CycloA* reference gene. Values represent averages of three technical replicates.

*SMCHD1* mutations promote aberrant inclusion of both exons 5 and 21-22 of DNMT3B, and *DNMT3B* mutations were previously associated with *DUX4* expression and D4Z4 hypomethylation (*19*). Thus, we next aimed to test whether *DNMT3B* alternative splicing can explain the D4Z4 hypomethylation and *DUX4* expression. To assess the influence of individual DNMT3B isoforms on D4Z4 methylation in a clinically relevant cell type for FSHD, we transduced human skeletal myoblasts with GFP-DNMT3B1, GFP-DNMT3B3ΔEx5, or an empty GFP vector (Fig. 8, A and B, and fig. S7, A to C). We specifically chose LHCN-M2 myoblasts that have a 4qA allele (fig. S7D). We determined DNA methylation levels after 3 days at the D4Z4 array using bisulfite-PCR sequencing and found a significantly lower D4Z4 methylation in DNMT3B1-expressing cells compared to DNMT3B3ΔEx5 cells (*P* < 0.008, Student’s *t* test) (Fig. 8, C and D). Next, we assessed the expression level of DUX4 mRNA (*39*) and found significantly higher levels of total DUX4 in DNMT3B1-expressing cells compared to DNMT3B3ΔEx5-expressing cells (2.9-fold enrichment, *P* < 0.016, Student’s *t* test; Fig. 8E and fig. S8A). To measure specifically the active and polyadenylated form of DUX4, we assessed its expression by qPCR using DUX4 full-length (DUX4-fl) isoform–specific primers (*40*) and found significantly higher levels in DNMT3B1-expressing cells compared to DNMT3B3ΔEx5-expressing cells (fivefold enrichment, *P* < 0.04, Student’s *t* test; Fig. 8F). Moreover, we determined DUX4 polyadenylated transcript levels using Oligo-dT–specific reverse transcription followed by qPCR and semiquantitative PCR. We found significantly higher levels of DUX4 and specifically DUX4-fl polyadenylated transcripts in DNMT3B1-expressing cells, while DNMT3B3ΔEx5-expressing cells had *DUX4* expression below detection rate (fig. S8, B and C). In addition, to assess DUX4 downstream activity, we measured the expression levels of four known DUX4 targets (*40*, *41*)—*ZSCAN4*, *PRAMEF1*, *DEFB103*, and *TRIM43*—and found significantly higher expression levels of all in DNMT3B1-expressing cells compared to DNMT3B3ΔEx5-expressing cells (1.35-, 11.5-, 1.7-, and 2.3-fold enrichment, respectively, *P* < 0.036, *P* < 0.0007, *P* < 0.025, *P* < 0.011, Student’s *t* test; Fig. 8G). These results demonstrate that *DNMT3B* alternatively spliced isoforms differentially regulate DNA methylation levels at the D4Z4 locus and *DUX4* expression and can therefore promote FSHD2 pathogenesis in skeletal myoblasts. Together with our finding that SMCHD1 mutations shift *DNMT3B* splicing toward the DNMT3B1 isoform, this suggests that *SMCHD1* mutations drive FSHD2 pathogenesis by altering *DNMT3B* splicing and prompting D4Z4 hypomethylation and *DUX4* expression.

Moreover, to assess the effect of *DNMT3B* mis-splicing on *DUX4* expression in undifferentiated cells, we overexpressed DNMT3B1 or DNMT3B3ΔEx5 in human embryonic stem cells without DNMT3B (DNMT3B-KO hESC). We assessed the expression level of DUX4 mRNA by qPCR and found robust expression of *DUX4* in DNMT3B1-expressing cells, but its expression in DNMT3B3ΔEx5-expressing cells was below the detection rate (Fig. 8H). This suggests that the shift in DNMT3B isoforms due to SMCHD1 loss can occur during different stages of differentiation, and the full effect in patients with FSHD2 is likely accumulated throughout development.

## DISCUSSION

**Fig. 9.**
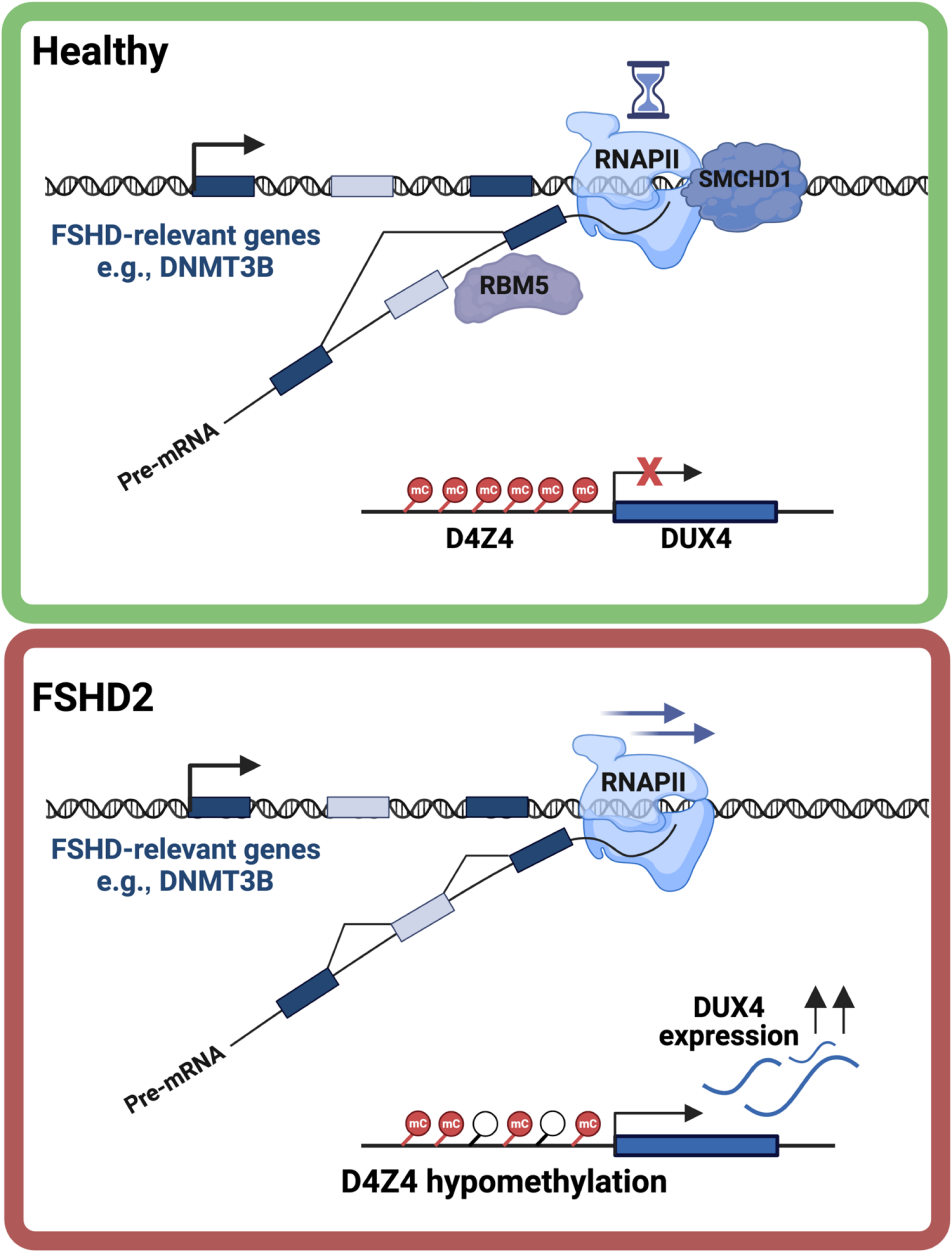
Model for SMCHD1 pathophysiology in FSHD2 driven by its abnormal splicing. In healthy cells, SMCHD1 binding is specifically enriched in the proximity of alternative exons, particularly excluded exons. A slower rate of RNAPII elongation is linked to SMCHD1 binding and related to exon exclusion. Excluded exons are characterized by a high density of RBM5 motifs, which inhibit exon inclusion and promote exon exclusion. However, in FSHD2 muscle cells, *SMCHD1* mutations lead to abnormal exon inclusion. This mis-splicing of FSHD-related genes, including *DNMT3B*, results in decreased methylation of the D4Z4 region and increased expression of the *DUX4* gene.

In this work, we demonstrate that SMCHD1 is an important splicing regulator and suggest a mechanism for its action. While SMCHD1 is known to regulate chromosomal interactions and gene expression, here, we show that it is also a splicing regulator. While previous reports did not detect differential isoform usage in Smchd1^MommeD1^ (*42*), here, we profiled mRNA by very deep sequencing accompanied by detailed differential spicing analysis allowing a more robust detection of exon inclusion and exclusion events. We suggest a mechanism in which SMCHD1 binding modulates RNAPII elongation rate. We hypothesize that RNAPII stalling is allowing the recruitment of inhibitory splicing factors and identify RBM5 as a potential ally in SMCHD1 regulatory pathway (Fig. 9).

Several chromatin modulators have previously been shown to act as regulators of splicing, and different mechanisms for chromatin-mediated splicing regulation were identified. Our results suggest that SMCHD1 regulation of splicing is mediated by RNAPII kinetics, similar to the mechanism suggested for CTCF-mediated splicing regulation (*5*, *6*). While CTCF is slowing RNAPII to promote exon inclusion, we found that SMCHD1-mediated slow RNAPII kinetics is correlating with exon exclusion (Fig. 3B) and is mediated by recruitment of splicing factors and specifically RBM5 (Fig. 6, A to D). Specifically, we show that SMCHD1 binds the vicinity of its target exons to stall RNAPII and allow for RBM5 binding to pre-mRNA (Figs. 2B, 3A, and 7, B and C). Overall, our results reveal a chromatin-mediated mechanism for splicing regulation.

We found SMCHD1 to regulate the splicing of multiple genes with a potential clinical significance for FSHD2 development. We found aberrant splicing of key factors for myocyte function whose disruption can contribute to muscular dystrophy. For example, *Titin (TTN)* is mis-spliced in patients with FSHD2. Mutations in *TTN* are a known cause for several muscular dystrophies (*43*) including limb-girdle muscular dystrophy (LGMD) and has many alternatively spliced isoforms with distinct functions (*44*), suggesting that splicing alteration of this gene can be associated with muscular pathology. Another example is the *calpain 3* (*CAPN3*) gene, which is mis-spliced in patients with FSHD2, and its mutations are causing LGMD as well (*45*). *CAPN3* was previously identified as significantly alternatively spliced in patients with FSHD1, and its alternative splicing resulted in muscle cells differentiation defect (*46*), suggesting that it has different functioning isoforms. We found that *CAPN3* is also differentially spliced between patients with FSHD1 and with FSHD2, at different exons from the previously reported one in FSHD1 (table S1). Specifically, the splicing alteration we identified in exons 15 and 16 was previously described to disrupt skeletal muscle mass (*47*). Moreover, we found significant alternative splicing changes in *Troponin T 1* (*TNNT1*) and *TNNT3* genes in patients with FSHD2. Both genes are key factors in myocyte function and have known alternatively spliced isoforms (*48*). *TNNT3* was previously shown as alternatively spliced in FSHD, and its aberrant splicing was found to characterize dystrophic muscles in patients with FSHD (*49*, *50*). Overall, the cumulative effect of these alterations and others we identified may contribute to the phenotype of FSHD and may potentially explain how SMCHD1 loss increases the clinical severity of patients with FSHD1 (*21*).

However, as FSHD1 is known to be caused directly by D4Z4 shortening and hypomethylation, and as FSHD1 and FSHD2 are clinically similar, it suggests that the primary effect of SMCHD1 loss is the inability to maintain D4Z4 methylation and *DUX4* silencing. We have therefore focused on *DNMT3B*, which contributes to D4Z4 methylation, and, therefore, perturbing it may mimic the cis effects of D4Z4 shortening, as observed in patients with *DNMT3B*-mutant FSHD4. Previous findings indicate that Dnmt3b is binding to the D4Z4 array, and its knockdown results in elevated *DUX4* expression in several cell types (*51*). While *SMCHD1* mutations are known to be present in patients with FSHD2 and are associated with D4Z4 hypomethylation and *DUX4* expression, the specific mechanism for SMCHD1-associated hypomethylation is unclear. Our findings suggest that mutations in *SMCHD1* lead to the mis-splicing of multiple genes including *DNMT3B*, supporting the DNMT3B1 isoform over the DNMT3B3ΔEx5 isoform (Fig. 4C). Last, *DNMT3B* mis-splicing causes D4Z4 hypomethylation and increase in *DUX4* expression in human skeletal myoblasts, suggesting that this effect also occurs in muscle cells of patients with FSHD2 (Fig. 8, C to F). The detected change in DNA methylation at D4Z4 is modest, yet the observed *DUX4* overexpression suggests that it is functional, likely representing a change in a subset of the cells and, therefore, a modest average methylation change. Together, our results suggest an updated and elaborate model for FSHD pathogenesis orchestrated by SMCHD1 (Fig. 9).

Beyond the direct effect of SMCHD1 on splicing regulation of SMCHD1 bounded genes, our results suggest a broader indirect impact potentially mediated by changes in splicing of several splicing factors (table S1). We did not detect differential expression of splicing factors upon loss of SMCHD1 in any of the cell types (except for Elavl2 in MEFs; see table S3), suggesting that modulation of mRNA levels of splicing factors does not play a role in this. However the mis-splicing of splicing factors may suggest that the overall impact of SMCHD1 loss on mis-splicing is greater than its direct involvement in splicing regulation and can explain the splicing changes of genes not bound by SMCHD1. Together, our findings shed light on the intricate interplay between SMCHD1 and the splicing machinery, adding a layer to its function in gene regulation.

Two *DNMT3B* mutations have been previously studied in the context of FSHD (*19*). While the specific role of *DNMT3B* variants in FSHD development is not fully clear, the described mutations have been shown to either disrupt the C2C2-type zinc-finger motif in ATRX-DNMT3-DNMT3L domain in exon 15 or reside in the C-terminal catalytic domain of DNMT3B in exon 19 and possibly alter its DNA methylation activity. In addition, biallelic *DNMT3B* mutations in Immunodeficiency, Centromeric instability, and Facial anomalies syndrome 1 (ICF1) patients, most of which are loss of function variants in the C-terminal catalytic domain of DNMT3B, are also associated with severe D4Z4 hypomethylation.

In our work, we present that the shift from the DNMT3B3ΔEx5 isoform, which does not contain the entire C-terminal catalytic domain, to the DNMT3B1 isoform, which does contain it, leads to D4Z4 hypomethylation. While the expected role of the “active” isoform is opposing the observed finding (*52*), we hypothesize that DNMT3B active-inactive isoform balance is important for keeping D4Z4 methylated by complexes involving DNMT3B. DNMT3B inactive isoforms were previously shown to enhance DNA methylation and were suggested as accessory proteins to recruit and positively regulate DNMT3A (*53*, *54*). Specifically, previous studies indicate a gain in Dnmt3a catalytic efficiency following the presence of Dnmt3b inactive isoforms and that Dnmt3a and Dnmt3b3 form a stable complex (*54*). Moreover, the reintroduction of DNMT3B inactive isoform constructs in DNMT3B null HCT116 cells was shown to cause genome-wide methylation restoration in a similar matter to that of DNMT3B1 active isoform (*53*). Thus, it may be possible that the absence of the inactive isoform, which plays a crucial cofactor in DNMT3 complex activity, leads to hypomethylation. Thus, mis-splicing and down-regulation of the inactive isoform may disrupt the methylation of D4Z4 and cause FSHD.

## MATERIALS AND METHODS

### Cell lines

HCT116 (American Type Culture Collection, CCL-247, RRID:CVCL_0291) cells were grown in Dulbecco’s modified Eagle’s medium supplemented with 10% fetal bovine serum. Lymphoblastoid cell lines were grown in RPMI 1640 supplemented with 20% fetal bovine serum. Immortalized human skeletal myoblasts (LHCN-M2) (*55*) were grown in a 1:1 mix of Ham’s F12 nutrient mixture and skeletal Muscle Cell Growth Medium (PromoCell) supplemented with 20% fetal bovine serum. DNMT3B knockout hESC (DNMT3b-KO clone CL19) were obtained and grown in feeder-free culture conditions using defined mTeSR medium on vitronectin (Gibco)–coated plates as recently described (*56*). Cell lines were maintained at 37°C and 5% CO_2_ atmosphere.

### RNA-seq and analysis

RNA from WT and Smchd1-null (Smchd1^MommeD1/MommeD1^) NSCs from embryonic day 14.5 (E14.5) mouse brains was obtained as previously described (*42*). We performed next-generation sequencing using the RNA ScreenTape kit (catalog no. 5067-5576; Agilent Technologies), and D1000 ScreenTape kit (catalog no. 5067-5582; Agilent Technologies), Qubit RNA HS Assay kit (catalog no. Q32852; Invitrogen), and Qubit DNA HS Assay kit (catalog no. 32854; Invitrogen) were used for each specific step for quality control and quantity determination of RNA and library preparation. For mRNA library preparation: TruSeq RNA Library Prep Kit v2 was used (Illumina). In brief, 1 μg was used for the library construction; the library was eluted in 20 μl of elution buffer. Libraries were adjusted to 10 mM, and then 10 μl (50%) from each sample was collected and pooled in one tube. Multiplex sample pool (1.5 pM including PhiX 1.5%) was loaded in NextSeq 500/550 High Output v2 kit (75 cycles cartridge and 150 cycles cartridge; Illumina). Run conditions were in paired-end [43 × 43 base pair (bp) and 80 × 80 bp, respectively] and loaded on NextSeq 500 System machine (Illumina). NSC RNA-seq data were deposited in NCBI’s Gene Expression Omnibus and are accessible through GEO Series accession number GSE223039. FSHD patients’ RNA-seq data were obtained from the GEO database, accession GSE56787 (*32*). SMCHD1 mutant MEFs RNA-seq data were obtained from GEO, accession GSE121184 (*12*). SMCHD1 KO mESCs RNA-seq data were obtained from GEO, accession GSE126467 (*26*).

Reads were aligned to the mm10 (NSCs and MEFs) or hg38 (patients with FSHD) using STAR version 2.7.10 (*57*) with default parameters. We counted reads in genes with featureCounts version 2.0.1(*58*) using GENCODE release M21 (NSCs and MEFs) or GENCODE release 33 (patients with FSHD). For data of patients with FSHD, reads from biopsies of nine healthy controls, nine patients with FSHD1, and four with FSHD2 were analyzed. DESeq2 (*59*) was used to identify differentially expressed genes using FDR threshold of 0.05. rMATS (version 4.1.1) (*60*) was used to identify differential alternative splicing events. For each alternative splicing event, we used the calculation on both the reads mapped to the splice junctions and the reads mapped to the exon body [Junction Counts and Exon Counts (JCEC)] and filtered events by FDR threshold of 0.05 and inclusion level difference of 10% while also filtering out lowly expressed genes by requiring TPM > 1. Gene set enrichment analysis was performed using g:Profiler (*61*) online tool or the R package “gprofiler2” using the HPO (*35*) June 2022 release. To limit bias due to the inability to call differential splicing in lowly expressed genes, we limited the analysis only to genes with expression >1 TPM (table S3), comparing alternatively spliced genes with >1 TPM to all genes with >1 TPM. The FDR was controlled by the Benjamini-Hochberg procedure.

### ChIP-seq and analysis

Smchd1-GFP ChIP-seq data of NSCs were obtained from the GEO database, accession GSE111722 (*11*) and GSE174066 (*31*). Smchd1 ChIP-seq data of MEFs were obtained from the GEO database, accession GSE111820 (*12*). RNAPII phospoS2 ChIP-seq data of C2C12 cells and RNAPII ChIP-seq of MEFs were obtained from ENCODE (*29*), accessions ENCSR000AIU and ENCSR000CBX. For Smchd1 ChIP, FASTQ files were obtained using SRA toolkit (version 3.0.0) and aligned to mm10 genome with BWA-mem (version 0.7.17) (*62*) using default parameters. PCR duplicates were marked and removed using Samtools (version 1.15.1) (*63*).

Peak calling and annotation were performed using HOMER version 4.11 (*64*). We called Smchd1 peaks using the “histone” mode; otherwise, default parameters were used. Peak calling was done relative to the background signal using input for MEFs, whole-cell extract for the 2021 dataset of NSCs (GSE174066), and GFP ChIP-seq in WT cells for the 2018 dataset of NSCs (GSE111722). ChIP-seq signal estimation and visualization were done using HOMER annotatePeaks tool histogram mode, with the following parameters: -size 5000 -hist 50 -ghist. The average ChIP signal and scanning electron microscopy were calculated for each 50-bp genomic bin. The significance of differential binding was called using the Wilcoxon test with FDR correction by the Benjamini-Hochberg procedure.

### siRNA interference

HCT116 cells were seeded in six-well culture plates (1.75 × 10^5^ cells per well). After 24 hours, cells were transfected with 20 μM of esiRNA (Sigma-Aldrich) using TransITx2 system Mirus Bio (MIR2700, Thermo Fisher Scientific) following the manufacturer’s instructions. As a control, cells were transfected with esiRNA directed for GFP (EHUEGFP, Sigma-Aldrich). For each condition, cells were seeded as triplicates and collected for examination after 72 hours.

### CPT treatment

To impede the dynamics of transcribing RNAPII, HCT116 cells were plated to achieve 60% confluency and treated with CPT (Sigma-Aldrich) to a final concentration of 6 μM for 6 hours. As a control, cells were treated with dimethyl sulfoxide at the same concentration.

### qRT-PCR

RNA was isolated using the GENEzol TriRNA Pure Kit (Geneaid) or QuickExtract solution (Lucigen) for hESC samples. With the qScript cDNA Synthesis Kit (Quantabio), cDNA was synthesized from 1 μg RNA in a 20-μl reaction volume and afterward diluted to 4 ng/μl. For a specific reverse transcription of poly-adenylated transcripts, we used the qScript Flex cDNA Synthesis Kit (Quantabio) with Oligo-dT primers. For quantitative real-time PCR, 20 ng of cDNA and 1 pmol/μl of primers were mixed with 2× SYBR (Bio-Rad) in a total volume of 13 μl for each well. Cyclophilin A was used as a reference gene. Reactions were performed for 40 cycles with a *T*_m_ of 60°C. Values of technical triplicates were averaged. Primers used in this study are provided in table S4.

### Semiquantitative PCR

RNA was isolated using the GENEzol TriRNA Pure Kit (Geneaid). With the qScript cDNA Synthesis Kit (Quantabio), cDNA was synthesized from 1 μg of RNA in a 20-μl reaction volume and afterward diluted to 4 ng/μl. For a specific reverse transcription of poly-adenylated transcripts, the qScript Flex cDNA Synthesis Kit (Quantabio) was used with Oligo-dT primers. The PCR reaction was done with Taq Mix Red PCR MasterMix (Tamar), 40 ng of cDNA, and 1 pmol/μl of primers.

### ChIP-qPCR

Approximately 1 × 10^7^ HCT116 cells per sample were cross-linked for 10 min in 1% formaldehyde at room temperature and quenched with 0.1 M glycine. Cells were washed twice with cold phosphate-buffered saline (PBS) and lysed with lysis buffer [0.5% SDS, 10 mM EDTA, 50 mM tris-HCl (pH 8), and protease inhibitor]. DNA was sonicated in an ultrasonic bath (Diagenode, Bioruptor) to an average length of 300 to 500 bp. Supernatants were immunoprecipitated overnight with RNAPII pS2 antibody (Abcam, Ab5095, RRID:AB_304749). Protein G beads (Cell Signaling Technology, no. 9006) were added to immunoprecipitated samples and incubated for 2.5 hours at 4°C. The beads were washed sequentially for 5 min each in low-salt radioimmunoprecipitation assay [RIPA: 20 mM tris-HCl (pH 8), 150 mM NaCl, 2 mM EDTA, 1% Triton X-100, and 0.1% SDS], high-salt RIPA [20 mM tris-HCl (pH 8), 500 mM NaCl, 2 mM EDTA, 1% Triton X-100, and 0.1% SDS], LiCl buffer [10 mM tris (pH 8.0), 1 mM EDTA, 250 mM LiCl, 1% NP-40, and 1% Na-deoxycholate], and in tris-EDTA buffer. Beads were eluted in TE (pH 8.0), 0.1% SDS, and 150 mM NaCl with 1:200 dithiothreitol 1 M buffer for 1 hour at 65°C, followed by ribonuclease treatment for 30 min at 37°C. Proteinase K was added and incubated at 50°C for 2 hours. Reverse cross-linking was carried out by adding NaCl to a final concentration of 200 mM and incubation for 2 hours at 65°C. DNA was extracted using magnetic beads (Beckman Coulter, Agencourt AMPure XP, catalog no. A63881). Immunoprecipitated DNA (2 of 50 μl) and serial dilutions of input DNA (1:5, 1:2.5, 1:1.25, and 1:0.625) were analyzed by real-time qPCR. ChIP-qPCR data were analyzed relative to input to include normalization for both background levels and the amount of input chromatin to be used in ChIP.

### siRNA splicing factor screen

EsiRNA library targeting 71 human splicing factors was purchased from Sigma-Aldrich. HCT116 cells were seeded in a 96-well plate and reverse transfected with 50 nM of esiRNA using the Mirus TransITx2 system. We used siSMCHD1 as the positive control and siGFP (Sigma-Aldrich) as a negative control. After 72 hours, the cells were lysed with Bio-Rad’s SingleShot buffer. The cell lysate was used directly for cDNA synthesis followed by qPCR. PSI fold change values were calculated for DNMT3B exon 5 and exon 21 by DNMT3B exon inclusion/DNMT3B total mRNA and normalized to siGFP for each experiment individually to normalize the variance between experiments. *Z* scores were calculated for average PSI fold change values (averaged between the two biological replicates).

### Lymphoblastoid cell line establishment

Human samples were obtained under the Hadassah Institutional Helsinki committee, approval no. 0198-11 HMO. All patients gave an informed consent. Venous blood was collected in EDTA-coated tubes. Peripheral blood mononuclear cell separation was performed using Lymphoprep (Stemcell Technologies). Cells were incubated with B95 cell line media containing Epstein-Barr virus, and cyclosporine was added for 7 days of incubation. SMCHD1 protein levels were measured by Western blot using A302-872A (Bethyl Laboratories) antibody.

### RNA immunoprecipitation

Cells were washed with ice-cold PBS, harvested, and lysed for 30 min on ice in a buffer containing 0.5% NP-40, 150 mM NaCl, 50 mM tris-HCl (pH 7.5), and 1 mM EDTA supplemented with protease and RNasin inhibitors followed by sonication in an ultrasonic bath (Qsonica, Q800R2 Sonicator) for six cycles of 5-s ON and 30-s OFF. Supernatants were collected after centrifugation at 16,000*g* for 20 min. Antibodies with beads were incubated for 2 hours at 4°C. Sonicated lysate with preincubated beads was added for an additional 4 hours. Beads were washed four times, and GeneZol was added for RNA extraction. Serial dilutions of the 10% input cDNA (1:2, 1:10, 1:50, and 1:250) were analyzed by real-time qPCR. The oligonucleotide sequences used are listed in table S4. RBM5 levels were monitored by Western blot using anti-RBM5 antibody (Abcam, ab245646). β-Actin levels were assessed using anti–β-ACTIN antibody (Cell Signaling Technology, no. 4967).

### 4q35 haplotype sequencing

DNA was extracted from LHCN-M2 cells using the NucleoSpin Tissue kit (Macherey-Nagel). PCR was performed with previously described primers (*65*) for the 4q35 region. The PCR reaction was done with Taq Mix Red PCR MasterMix (Tamar). The PCR product was cleaned and sequenced by Sanger sequencing using the forward primer. Single-nucleotide polymorphisms were analyzed compared to previously described 4q35 analysis (*66*).

### DNMT3B isoforms overexpression

pLenti cytomegalovirus (CMV) GFP Puro (Addgene, plasmid no. 17448) plasmid was cut with BsrgI and SalI restriction enzymes. Sequences of DNMT3B transcripts were isolated from pcDNA4-DNMT3B1 and pcDNA4-DNMT3B3ΔEx5 plasmids (*36*) by PCR and assembled with pLenti construct using Gibson assembly (New England Biolabs) following the manufacturer’s protocol.

Lentiviruses were produced by transfection in the human embryonic kidney 293T packaging cell line using the PEI transfection reagent. Cells were transfected with each DNMT3B or GFP-only construct in addition to pCMV-dR8.2 dvpr (Addgene, plasmid no. 8455) and pCMV vesicular stomatitis virus glycoprotein (Addgene, plasmid no. 8454) lentiviral particles. Immortalized human skeletal myoblasts were infected with viral particles and monitored for GFP expression; cells were incubated for 3 days following infection. The number of GFP-expressing cells for each infection was assessed using the “analyze particles” tool of ImageJ with multiple images per condition. DNMT3B-KO hESCs were plated 1 day before infection at 3 × 10^5^ per six wells. One milligram of virus was concentrated with lentivirus concentrator and resuspended on mTeSR medium, and polybrene (8 μg/μl) was added. Cells were replaced with fresh mTeSR each day for 4 days starting 24 hours after infection. GFP fluorescence was followed by microscope and after 4 days sorted by fluorescence-activated cell sorting (FACS) using FacsAria III BD machine.

### Bisulfite sequencing and analysis

DNA was extracted from LHCN-M2 cells using the NucleoSpin Tissue kit (Macherey-Nagel). Bisulfite conversion was performed using Zymo MethylGold kit following the manufacturer’s protocol. PCR reaction to isolate the D4Z4 region was done by using previously described primer sets (*67*) containing Illumina adaptor sequences at both the 5′ and 3′ ends. The PCR reaction was done with Taq Mix Red PCR MasterMix (Tamar). PCR products were cleaned and directly amplified using primers for the Illumina adaptor sequences followed by DNA sequencing. Methylation analysis was done using Biscuit (version 1.0.2). Reads were aligned using Biscuit align to the GRCh38 genome. The methylation level was estimated using Biscuit pileup with the -m 0 parameter, followed by the biscuit vcf2bed tool with the -t cg parameter. Otherwise, default parameters were used.

### Motif enrichment analysis

RNA binding protein motif enrichment was performed using the SEA tool from the MEME suite (version 5.5.0) (*68*). Enrichment analysis was performed for excluded exon sequences with included exon sequences as background for human FSHD and mouse NSC results separately. FASTA files were generated using BEDTools getfasta tool (version 2.30.0) (*69*) with hg38 and mm10 reference genomes, respectively. SEA analysis was performed using default parameters and a default E value of ≤10 using the motif database: Ray2013 RNA (DNA-encoded) (*70*).
