# Supplementary material for: DNMT3B splicing dysregulation mediated by SMCHD1 loss contributes to DUX4 overexpression and FSHD pathogenesis
Source: Sci Adv. 2024 May 29;10(22):eadn7732. doi: 10.1126/sciadv.adn7732 (PMC11135424; doi:10.1126/sciadv.adn7732)
Supplement: Supplementary file 1 — Figs. S1 to S8 Legends for tables S1 to S4 [file sciadv.adn7732_sm.pdf]

Science Advances
AAAS

Supplementary Materials for

DNMT3B splicing dysregulation mediated by SMCHD1 loss contributes to DUX4 overexpression and FSHD pathogenesis

Eden Engal *et al.*

Corresponding author: Maayan Salton, maayan.salton@mail.huji.ac.il; Yotam Drier, yotam.drier@mail.huji.ac.il

Sci. Adv. **10**, eadn7732 (2024)
DOI: 10.1126/sciadv.adn7732

The PDF file includes:

Figs. S1 to S8
Legends for tables S1 to S4

Other Supplementary Material for this manuscript includes the following:

Tables S1 to S4

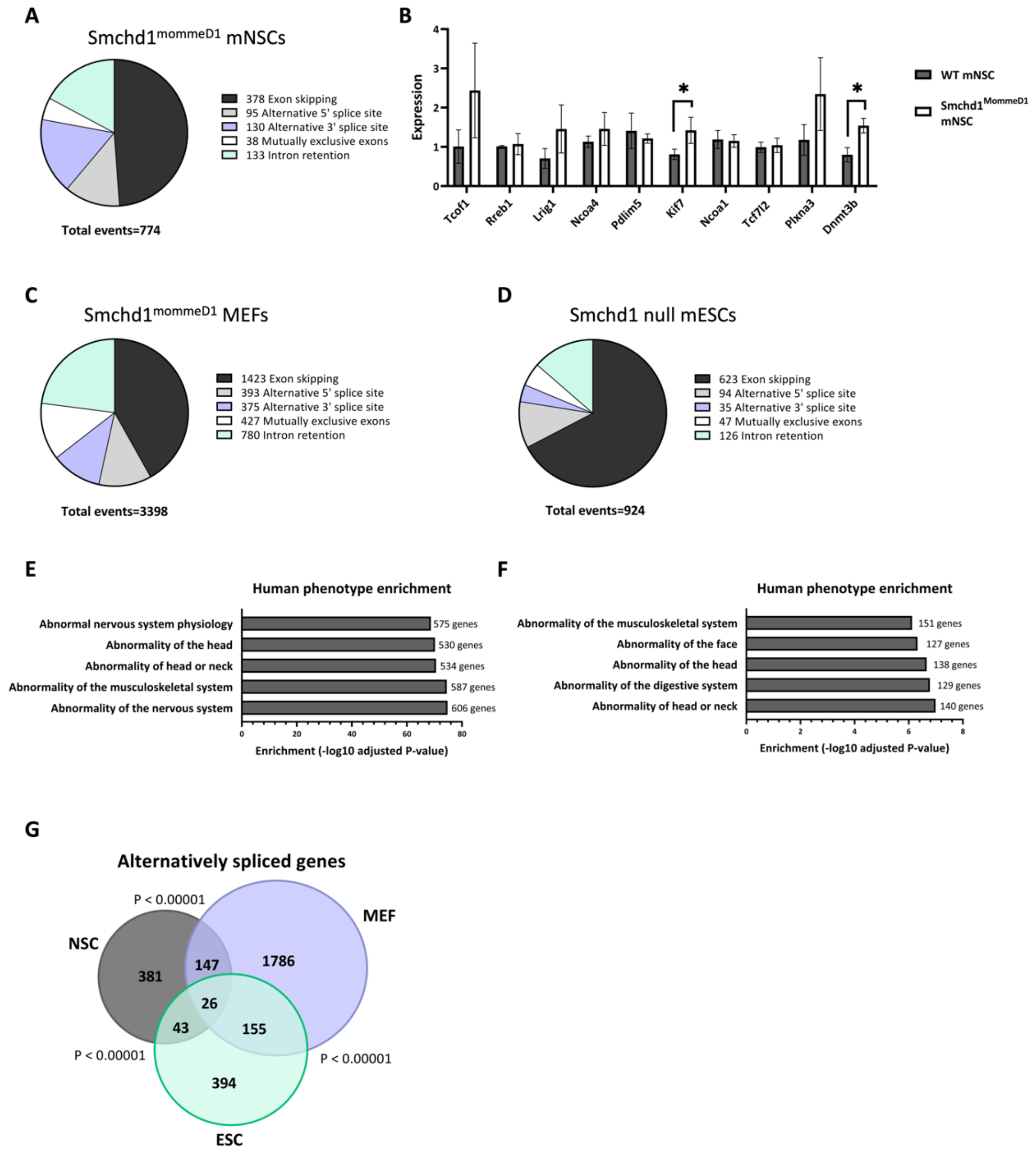

Fig. S1.

A. RNA was extracted from neural stem cells from three homozygous Smchd1^{MommeD1} and two wildtype mice and deeply sequenced. Significant alternative splicing events were detected by rMATS analysis. Pie chart shows the abundance of different types of significant alternative splicing events (FDR<0.05, PSI > |0.1|, TPM > 1). **B.** Real-time PCR was conducted to measure total mRNA amount. Values represent averages of three RNA samples relative to four control samples ±SD. * p<0.05. **C-D.** Pie charts show the abundance of different types of significant alternative splicing events (FDR<0.05, PSI > |0.1|, TPM > 1) in Smchd1^{MommeD1} MEFs **(C)** and Smchd1-KO mESCs **(D)** RNA-seq data. **E-F.** Five most significant human phenotype enriched with alternatively spliced genes in MEFs **(E)** and mESCs **(F)**, significance represented as -log10 adjusted p-value. **G.** Venn diagram presenting the overlap between Smchd1 alternative splicing regulation in mNSCs, mESCs and MEFs of Smchd1 null or Smchd1-KO mice.

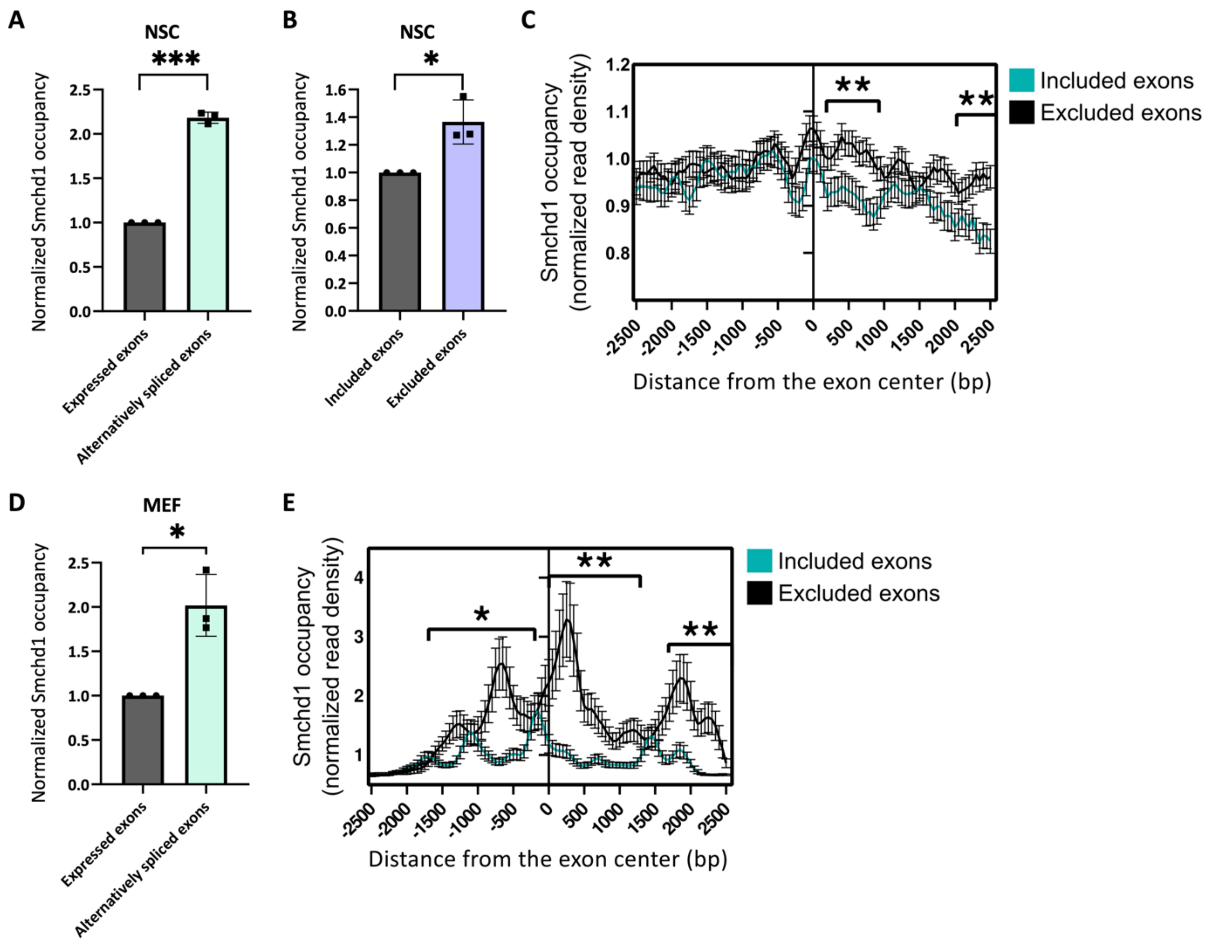

Fig. S2.

A-B. GFP ChIP-seq in primary NSCs with endogenous Smchd1-GFP fusion protein. Smchd1 occupancy analyzed as the number of Smchd1 peaks present within 5kb of alternatively spliced or expressed (TPM>1) exons **(A)** or within 5kb of differentially included or excluded exons in Smchd1^{MommeD1} NSCs **(B)**. Values represent averages of three ChIP-seq replicates from each cell type ±SD [*p<0.05; **p<0.01; ***p<0.001]. **C.** Aggregation plot depicting the average normalized Smchd1 occupancy, at and near exons differentially included or excluded in Smchd1^{MommeD1} NSCs. The x-axis represents bins of size 50 bp around the center of the exon [*p<0.05; **p<0.01]. **D.** SMCHD1 ChIP-seq in MEFs. SMCHD1 occupancy analyzed as the number of SMCHD1 peaks present within 5kb of alternatively spliced or expressed (TPM>1) exons in Smchd1^{MommeD1} MEFs. Values represent averages of three ChIP-seq replicates from each cell type ±SD [*p<0.05; **p<0.01; ***p<0.001] **E.** Aggregation plot depicting the average normalized Smchd1 occupancy, at and near exons differentially included or excluded in Smchd1^{MommeD1} MEFs. The x-axis represents bins of size 50 bp around the center of the exon [*p<0.05; **p<0.01].

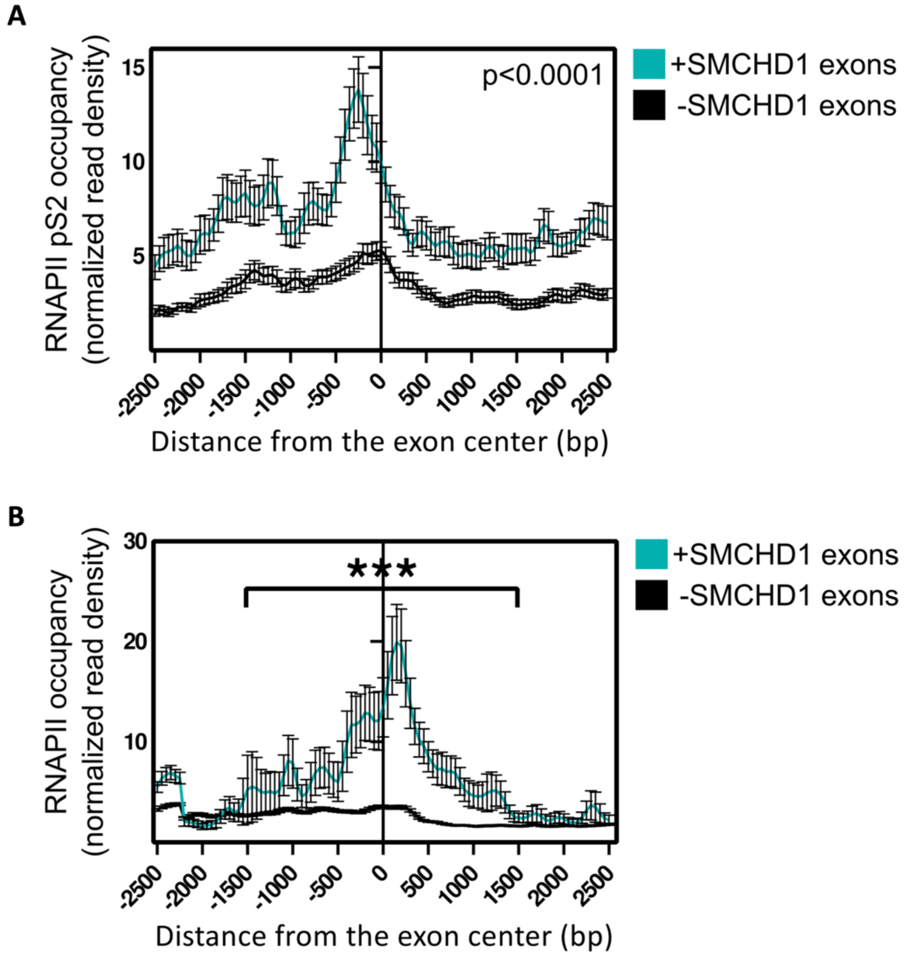

Fig. S3.

A. Aggregation plot depicting the average normalized phospho-Ser2 levels of RNAPII at and near alternatively spliced exons differentially bound by Smchd1 in NSCs. The x-axis represents bins of size 50 bp around the center of the exon. **B.** Aggregation plot depicting the average normalized levels of RNAPII (MEFs) at and near alternatively spliced exons differentially bound by Smchd1 in MEFs. The x-axis represents bins of size 50 bp around the center of the exon.

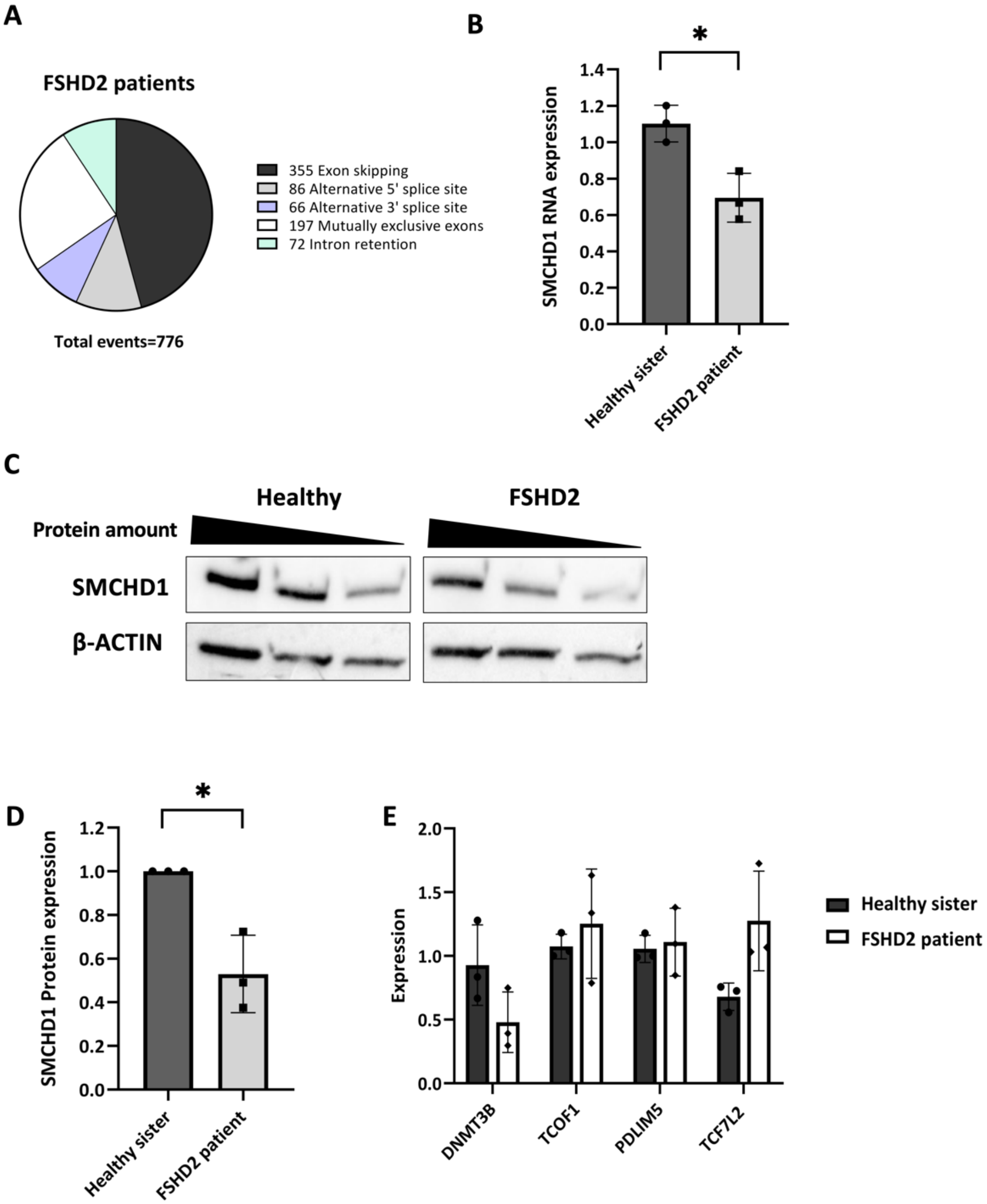

Fig. S4.

A. Pie chart shows the abundance of different types of significant alternative splicing events (FDR<0.05, PSI > |0.1|, TPM > 1) in muscles of FSHD2 patients compared to healthy controls as detected by rMATS analysis of RNA-seq data. **B-E.** RNA was extracted from lymphoblasts of an FSHD2 patient and her healthy sister and analyzed by real-time PCR for *SMCHD1* total mRNA amount relative to *CycloA* reference gene (**B**). Protein was extracted and Western blot was conducted with the indicated antibodies **(C**). Quantification of Western blot **(D)**. RNA was extracted and real-time PCR was conducted to the indicated gene relative to *CycloA* reference gene (**E**). Values represent averages of three experiments ±SD [*p<0.05].

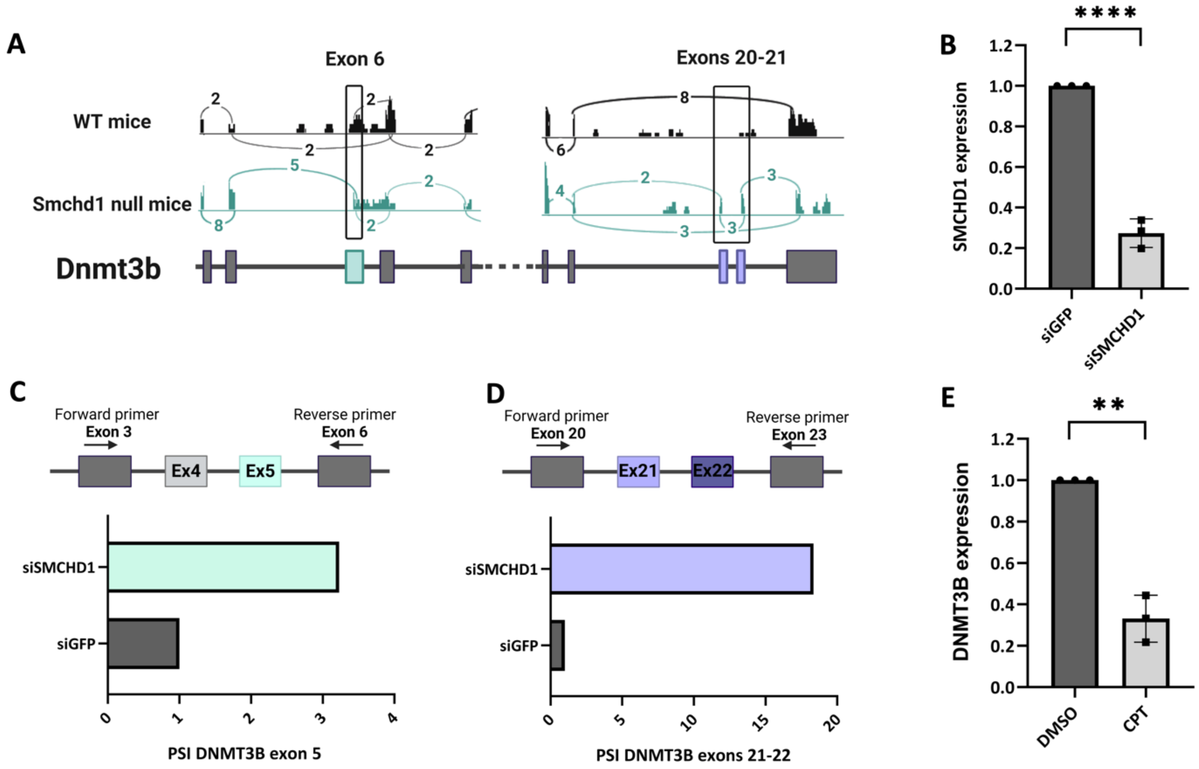

Fig. S5.
A. RNA was extracted from three Smchd1^{MommeD1} mouse NSC samples and two WT mouse NSC samples. RNA-seq was conducted and analyzed using rMATS (FDR<0.05). Genome browser view of the Dnmt3b alternatively spliced junctions presented by sashimi plots, arcs denote splice junctions quantified in spanning reads. **B-D.** HCT116 cells were transfected with siRNA targeting SMCHD1 or GFP as negative control. Total RNA was extracted and analyzed by real-time PCR for SMCHD1 total mRNA amount relative to *CycloA* reference gene. Values represent averages of three experiments done in triplicates ±SD normalized to negative control (siGFP) [****p<0.0001] (paired Student's t-test) **(B)**. Semi quantitative PCR was conducted for exons 4-5 **(C)** and exons 20-21 **(D)** using custom primers as described. PSI is calculated as the included product amount relative to the excluded product, and normalized to siGFP as a negative control. **E.** HCT116 cells were treated with 6uM of CPT or DMSO as negative control for 6 h. Total RNA was extracted and analyzed by real-time PCR for total DNMT3B mRNA amount relative to *CycloA* reference gene. Values represent averages of three experiments done in triplicates ±SD normalized to negative control (DMSO) [**p<0.01] (paired Student's t-test).

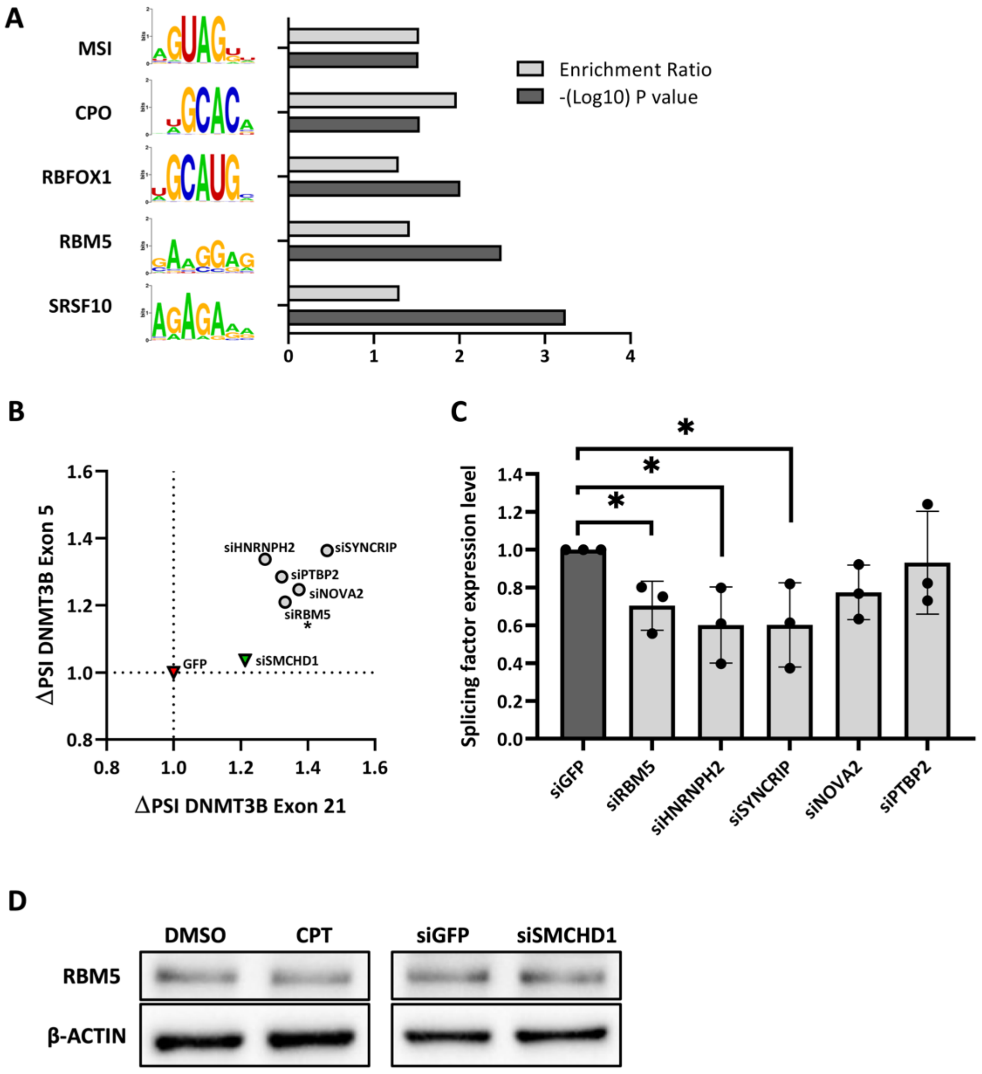

Fig. S6.

A. Enrichment of binding sites of SMCHD1 regulated exons: Enriched RNA motifs in alternatively excluded exons in both mouse and human compared to included exons. **B.** HCT116 cells were transfected with siRNA targeting 71 human splicing factors, SMCHD1 as a positive control and GFP as negative control. Total RNA was extracted and analyzed by real-time PCR for DNMT3B exon 5 and exon 21 relative to DNMT3B total mRNA amount. PSI was calculated as DNMT3B exon inclusion/DNMT3B total mRNA and normalized to negative control (siGFP). Values represent averages of two experiments ±SD. Negative control (siGFP) PSI is represented by the dotted line at 1. **C.** HCT116 cells were transfected with siRNA targeting each splicing factor hit indicated or GFP as a negative control. Total RNA was extracted and analyzed by real-time PCR for splicing factor total mRNA amount relative to *CycloA* reference gene. Values represent averages of three experiments done in triplicates ±SD normalized to negative control (siGFP) [*$p<0.05$] (paired Student's t-test). **D.** HCT116 were treated with 6uM CPT or DMSO as negative control, for 6 h (left panel) or transfected with siSMCHD1 or negative control (siGFP) for 72 h (right panel). Immonubloting for RBM5 was perfomed to monitor its expression and b-ACTIN was used as a control.

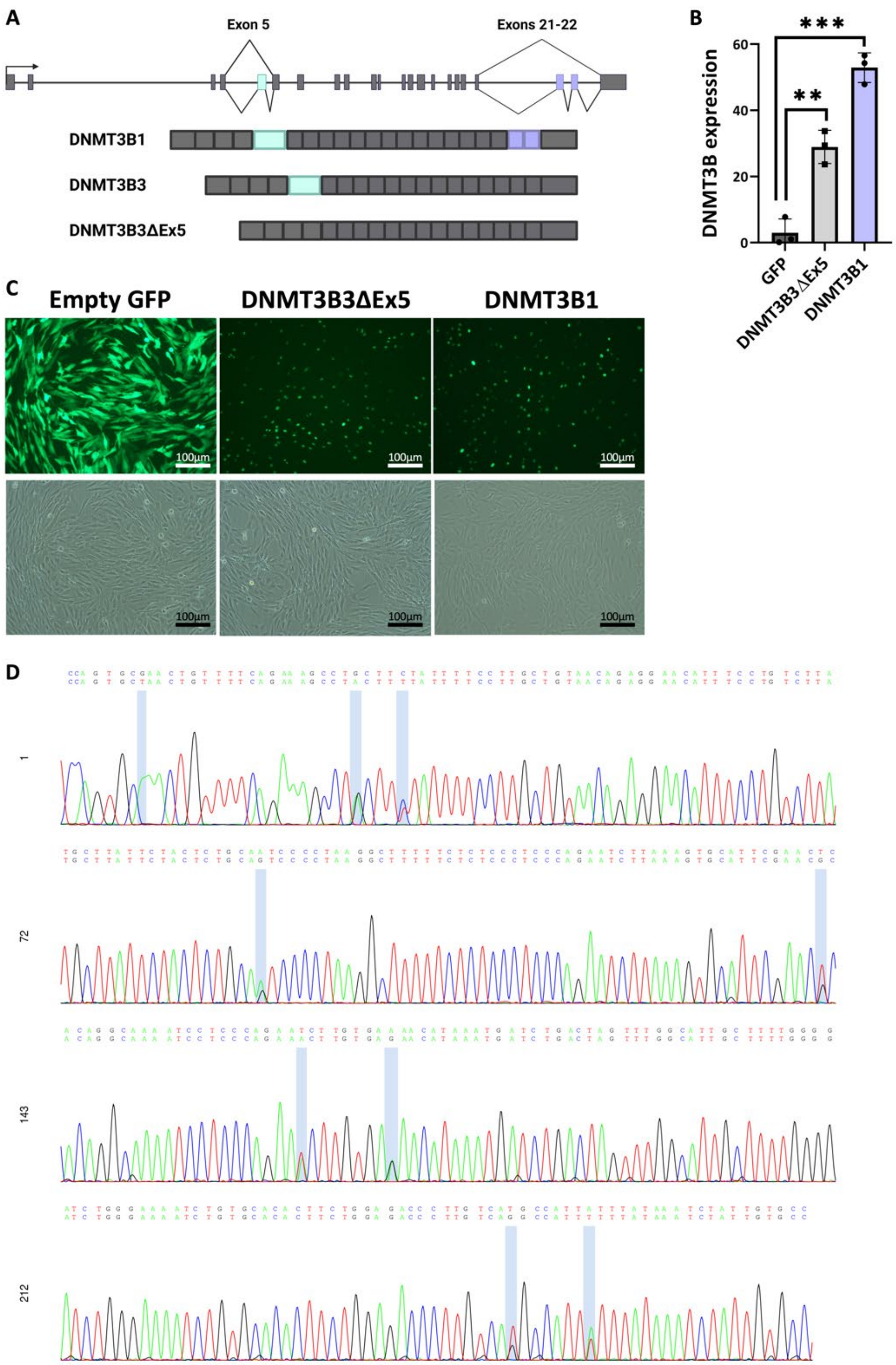

Fig. S7.
A. Schematic representation of the *DNMT3B* gene and its alternatively spliced isoforms. **B.** LHCN-M2 cells were infected with lentiviruses containing empty-GFP, GFP-DNMT3B3ΔEx5 or GFP-DNMT3B1. To assess successful infection, Real-time PCR was conducted and DNMT3B mRNA level was quantified relative to *CycloA* reference gene. Values represent averages of three technical replicates ±SD [** $p<0.01$; *** $p<0.001$] (Student's t-test). **C.** Snapshots of human skeletal myoblasts following infection with DNMT3B isoforms. ***D.*** Sanger sequencing of 4q35 array in LHCN-M2 cells. Monoallelic SNPs are highlighted in light blue, suggesting a single copy of the 4qA allele in the cells.

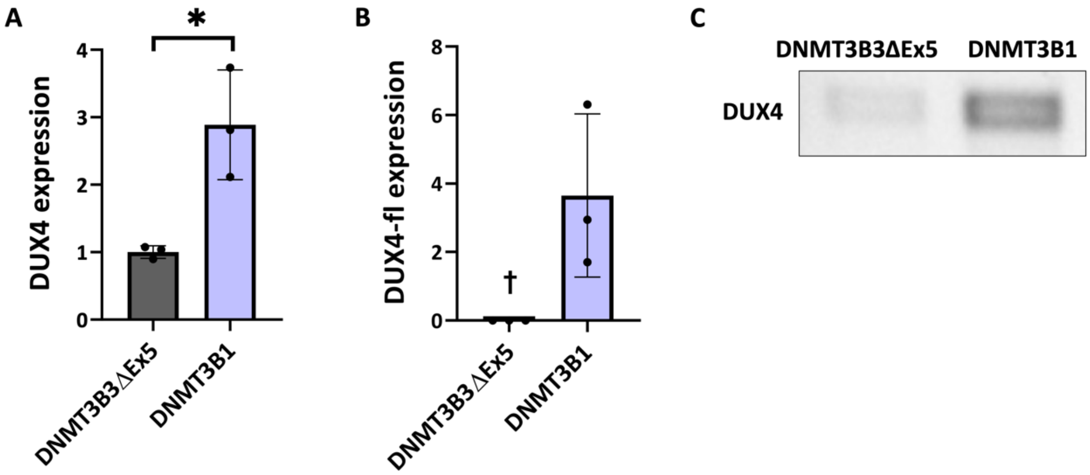

Fig. S8.
A. RNA was extracted from human skeletal myoblast cells with the DNMT3B3ΔEx5 and DNMT3B1 isoforms. RNA was reverse transcribed using random hexamer primer and real-time PCR was conducted for DUX4 mRNA. DUX4 mRNA level was quantified relative to *CycloA* reference gene. Values represent averages of three technical replicates ±SD [*$p<0.05$] (Student's t-test). **B.** RNA was reverse transcribed using oligo-dT specific primers and real-time PCR was conducted for DUX4-fl mRNA. DUX4-fl mRNA level was quantified relative to *CycloA* reference gene. Values represent averages of three technical replicates ±SD. † Expression level was below detection rate. **C.** RNA was reverse transcribed using oligo-dT specific primer and semi-quantitative PCR was conducted for DUX4 mRNA levels.

Legends for tables S1 to S4

Table S1. Significant alternative splicing events.

Table S2. Enrichment of human phenotypes in alternatively spliced genes.

Table S3. Gene expression levels and differentially expressed genes.

Table S4. Primers utilized in this study.
